# The genomic and transcriptome characteristics of lung adenocarcinoma patients with previous breast cancer

**DOI:** 10.1186/s12885-022-09727-6

**Published:** 2022-06-06

**Authors:** Yan Wang, Wenpeng Song, Sicheng Zhou, Shuai Chang, Junke Chang, Jie Tian, Liming Zhang, Jue Li, Guowei Che

**Affiliations:** Department of Thoracic Surgery, West China Hospital, Sichuan University, Guoxuexiang No. 37, Chengdu, 610041 China

**Keywords:** Breast cancer, Second primary, Lung adenocarcinoma, Genomic characteristics, Transcriptome characteristics

## Abstract

**Background:**

Breast cancer and lung cancer are the top two malignancies in the female population and the number of patients with breast cancer and subsequent primary lung cancer has increased significantly in recent years. However, the unique molecular characteristics of this group of patients remains unclear.

**Purpose:**

To identify the genomic and transcriptome characteristics of primary lung adenocarcinoma patients with previous breast cancer by comparison with single primary lung adenocarcinoma (SPLA) patients.

**Methods:**

The tumor and normal pulmonary tissue specimens of ten primary pulmonary adenocarcinoma patients with previous breast cancer (multiple primary cancer, MPC) and ten SPLA patients were prospectively collected. The whole exome sequencing (WES) and RNA sequencing (RNA-seq) were performed to analyze the gene mutation and expression differences between MPC and SPC patients.

**Results:**

The results of WES indicated that the mutations of TRIM73, DLX6 and CNGB1 only existed in MPC patients. The results of RNA-seq manifested the occurrence of second primary lung adenocarcinoma in breast cancer patients was closely associated with cytokine-cytokine receptor action, autophagy, PI3L-Akt, cAMP and calcium ion signaling pathways. Besides, the expression levels of FGF10 and VEGFA genes were significantly increased in MPC patients.

**Conclusion:**

The occurrence of second primary lung adenocarcinoma may be related to the cytokine-cytokine receptor action, autophagy, PI3L-Akt, cAMP and calcium ion signaling pathways. Furthermore, the mutations of TRIM73, DLX6 and CNGB1 and high expression of FGF10 and VEGFA might play an important role in the development of lung adenocarcinoma in breast cancer patients. However, more in-depth investigations are needed to verify above findings.

**Supplementary Information:**

The online version contains supplementary material available at 10.1186/s12885-022-09727-6.

## Introduction

Lung cancer and breast cancer are the two most common tumors worldwide, with more than two million new cases each year [1, 2]. Breast cancer is the most common malignant tumor and second cause of cancer-related mortality in women; meanwhile, lung cancer is the leading cause of cancer-related mortality and second most common malignancy in women [2]. In the past few decades, great progress in the early diagnosis and treatment of breast cancer have been made and the overall survival time of breast cancer patients has been significantly extended. On the other hand, the incidence of lung cancer has also continued to rise in the last decade [3]. Therefore, the number of breast cancer patients with subsequent primary lung cancer has increased obviously [4].

Actually, the risk of lung cancer in breast cancer patients has been verified to be higher than that in the general population [5]. Meanwhile, our previous research also identified several risk factors for second primary lung cancer after treatment of breast cancer. In detail, we demonstrated that the smoking [odds ratio (OR) = 9.73, *P *< 0.001] and radiotherapy [relative risk (RR) = 1.40, *P* < 0.001] were high-risk factors for developing second lung cancer in breast cancer patients and the chemotherapy (RR = 0.69, *P* = 0.002), positive estrogen receptor (ER) status (RR = 0.93, *P* = 0.014) and positive progesterone receptor (PR) status (RR = 0.86, *P* < 0.001) were protective factors for second primary lung cancer [5]. However, most of relevant researches about lung cancer after breast cancer are clinical retrospective observation studies up to now and few scholars focused on the molecular mechanisms of second primary lung cancer after treatment of breast cancer. Thus, the molecular characteristics of this group of patients remain unclear now, especially the unique genomic and transcriptome characteristics, which severely limits the effective clinical screening, management and intervention for these patients.

The aim of the current study was to identify the genomic and transcriptome characteristics of primary lung adenocarcinoma patients with previous breast cancer by comparison with single primary lung adenocarcinoma (SPLA) patients using the whole exome sequencing (WES) and RNA sequencing (RNA-seq).

## Materials and methods

### Ethical requirements

This study was performed according to the ethical standards of the national research committee and the 1964 Helsinki Declaration and its later amendments or comparable ethical standards. Meanwhile, this study was approved by the regional committee of Sichuan University West China Hospital (ID: 2020–250).

### Patient selection

#### Inclusion and exclusion criteria for multiple primary cancer (MPC) group

The following inclusion criteria were applied: 1) patients were pathologically diagnosed with primary breast cancer and received the surgical therapy; 2) female patients aged 18 to 80 years, without the history of smoking; 3) pulmonary nodules or masses were found after the breast cancer operation and the pulmonary tumor resection was performed at the Department of Thoracic Surgery of our hospital; 4) pulmonary nodules or masses were pathologically diagnosed with primary lung adenocarcinoma; 5) enough pulmonary adenocarcinoma and normal pulmonary tissue specimens for WES and RNA-seq; 6) complete clinicopathological and therapy-related data; 7) patients signed the informed consent.

The following exclusion criteria were applied: 1) combined with the history of other malignant tumors or genetic diseases; 2) unqualified sequencing data due to the specimen contamination, improper storage or insufficient cell numbers, etc.

#### Inclusion and exclusion criteria for single primary lung cancer (SPLC) group

The following inclusion criteria were used: 1) patients received the pulmonary resection for pulmonary nodules or masses at the Department of Thoracic Surgery in our hospital; 2) pulmonary nodules or masses were pathologically diagnosed with primary lung adenocarcinoma; 3) female patients aged 18 to 80 years, without the history of smoking; 4) enough pulmonary adenocarcinoma and normal pulmonary tissue specimens for WES and RNA-seq; 5) complete clinicopathological and therapy-related data; 6) patients signed the informed consent.

The following exclusion criteria were used: 1) combined with the history of other malignant tumors or genetic diseases; 2) unqualified sequencing data due to the specimen contamination, improper storage or insufficient cell numbers, etc.

### Surgical specimen collection

The fresh lung adenocarcinoma and normal pulmonary tissue specimens of ten MPC patients and ten SPLA patients who received the pulmonary tumor resection from March 2020 to July 2020 at the Department of Thoracic Surgery, West China Hospital were prospectively collected. The size of each sample should be at least 0.3 cm. The samples were obtained in the operating room and would be quickly frozen in liquid nitrogen and stored in the refrigerator at − 80 °C to ensure the integrity of the DNA and RNA.

### DNA/RNA co-extraction

The DNA/RNA co-extraction was performed by the AllPrep DNA/RNA Mini Kit which was used to purify high-quality DNA and RNA from single cells or tissue samples at the same time. This kit mainly uses the new AllPrep DNA spin column to purify the genomic DNA and then the AllPrep column effluent is purified by the RNeasey MinElute spin column to obtain total RNA, so as to achieve the purpose of co-extraction. The specific steps have been described in the supplementary file 1.

### Whole exome sequencing (WES)

The detailed equipment used for WES have been introduced in the supplementary file 1. The WES included the DNA quantification and detection, DNA fragmentation, end repair, 3’end with “A” tail, ligation of sequencing adapter, library fragment screening, DNA library amplification by polymerase chain reaction (PCR), hybrid capture, elution, purification, exon DNA library amplification by PCR, purification, library quality control, bridge PCR, library quantification and PE150 sequencing through the Illumina HiseqX TNN platform.

### RNA-sequencing

The RNA-seq included the RNA quantification and detection, library construction, quality control, cluster generation and sequencing through the Illumina-Hiseq platform. The specific process has been described in the supplementary file 1.

### Sequencing data quality control

For the WES, we finely filtered the raw reads and removed linkers and low-quality bases (< 20) in reads to obtain clear reads. The sequencing data quality assessment included the total base (> 8Gb), mapped ratio (> 98%), duplicated ratio (> 65% for tumor tissues and > 43% for normal pulmonary tissues), on-target ratio (> 40%), mean coverage (> 80), Q20% (> 85%) and Q30% (> 85%).

For the RNA-seq, after obtaining the raw data, we filtered the data including removing some low-quality reads (bases with Q ≤  20 accounted for more than half of the entire reads) and reads with connectors or a N ratio of more than 5% to ensure the quality and reliability of the data. After the filtering, clean reads were obtained and then we used the FASTQC software to evaluate the quality of clean reads. The evaluation indicators included the data volume, total mapped ratio (> 90%), uniquely mapped ratio (>80%), duplicated ratio (< 20%), OD 260/280 (> 1.50), Q20% (> 85%) and Q30% (> 85%). Meanwhile, the principal component analysis (PCA) was also performed to determine the level of repeatability between samples in different groups [6].

Only samples with qualified data quality could be further analyzed.

### Data analysis

The analysis for WES mainly focused on the variants including the single nucleotide variant (SNV), insertion and deletion (InDel) mutations, somatic copy number variation (SCNV), mutation spectrum analysis, microsatellite instability (MSI), tumor mutation burden (TMB) and high-frequency gene mutation analysis. The SNV and InDel mutation analyses were performed by comparing the database sequence to the human reference genome, using sentieon’s TNScope process to detect SNV and InDel mutations in lung tumor samples and normal lung tissue samples, and performing statistics, annotations and filtering. The CNV analysis was conducted by using CNVkit and the company’s self-developed EulerCNV program to detect the CNV information in lung tumor samples and normal lung tissue samples, and to complete statistics, annotations and filtering. The MSI was directly calculated by msisensor and was defined as the microsatellite stability (MSS: MSI < 20), moderate MSI (MSI-M: 20 ≤  MSI < 40), high MSI (MSI-H: 40 ≤  MSI < 60) and very high MSI (MSI-VH: MSI ≥ 60). Meanwhile, the TMB value was obtained by using the driver gene results output by SNV and was defined as the low TMB (TMB-L: TMB < 20), moderate TMB (TMB-M: 20 ≤ TMB < 40), high TMB (TMB-H: 40 ≤ TMB < 60) and very high TMB (TMB-VH: TMB ≥ 60).

For the RNA-seq, the Gene Set Enrichment Analysis (GSEA) was performed and the differentially expressed genes were defined as genes with false discovery rate (FDR)< 0.05 and a fold change of more than two times. GSEA used a predefined gene set to combine genes with the same or similar functions and encapsulates them in the form of a gene set. Then the differentially expressed genes in the test group and the control group were sorted to test whether the differentially expressed genes in the two groups were enrichment at the end or top of the predefined gene set. If it was enriched at the top, it meant that the gene set was generally up-regulated; and if it was enriched at the tail, it was down-regulated. In this study, the hallmark gene sets (h.all.v7.2.symbols.gmt), GO gene sets (c5.all.v7.2.symbols.gmt) and curated gene sets (Kyoto Encyclopedia of Genes and Genomes, KEGG) (c2.cp.kegg.v7.2.symbols.gmt) were applied as predefined gene sets to conduct the GSEA [7]. Several indicators were used as key statistics during the GSEA including the enrichment score (ES) which represented the degree of enrichment of a certain gene set in the two segments of the sorted list, normalized enrichment score (NES) which was obtained by normalizing the calculated ES according to the size of the gene set, FDR which indicated the estimated probability of false positive discovery and nominal *P* value was used to describe the statistical significance of ES derived from a functional gene set. Besides, the leading edge analysis was also conducted to further analyze the enriched functional gene sets obtained in the previous analysis and to explore whether there was overlap between the leading edge genes in these gene sets.

## Results

### Basic characteristics of included patients

Ten MPC patients and ten SPLA patients were enrolled in this study. No significant difference in the age was observed between the two groups and all patients were pathologically diagnosed with TNM stage I. The median time between the diagnosis of breast cancer and lung adenocarcinoma in the MPC group was 36 months, ranging from 6 to 90 months. The other basic information was presented in the Table 1.

**Table 1:** Basic characteristics of included patients in the whole exome sequencing

ID	Age (lung)	Age (breast)	Interval (months)	Family history of malignant tumors	TNM stage (lung)	Location (lung)	TNM stage (breast)	Location (breast)	ER	PR	HER-2	Chemotherapy	Radiotherapy
2,000,808	49	48	15	no	IA	right	IIIC	left	+	+	2+	yes	yes
2,001,331	44	41	40	no	IA	left	IA	right	+	+	2+	yes	no
2,001,347	45	44	10	yes	IA	right	IA	left	+	+	–	no	no
2,001,364	53	45	90	no	IA	left	IA	left	±	–	–	yes	no
2,001,589	31	31	6	no	IA	left	IA	left	+	+	3+	no	no
2,001,704	46	45	7	no	IA	right	IA	left	+	+	3+	yes	no
2,001,705	50	46	44	no	IA	left	IIA	left	–	–	+	no	no
2,002,120	66	61	60	yes	IA	left	IIA	right	–	–	3+	yes	no
2,002,259	55	53	28	no	IA	right	IIIC	left	+	–	3+	yes	yes
2,002,297	64	61	42	no	IA	right	IA	right	–	–	2+	yes	no
2,001,631	52	–	–	no	IA	left	–	–	–	–	–	–	–
2,001,701	70	–	–	no	IA	right	–	–	–	–	–	–	–
2,001,703	54	–	–	no	IA	right	–	–	–	–	–	–	–
2,002,031	57	–	–	yes	IA	left	–	–	–	–	–	–	–
2,002,191	48	–	–	no	IA	left	–	–	–	–	–	–	–
2,002,298	44	–	–	yes	IA	right	–	–	–	–	–	–	–
2,002,388	67	–	–	no	IA	right	–	–	–	–	–	–	–
2,002,414	55	–	–	no	IA	left	–	–	–	–	–	–	–
2,002,560	68	–	–	no	IA	left	–	–	–	–	–	–	–
2,002,561	64	–	–	no	IA	right	–	–	–	–	–	–	–

*TNM* tumor-node-metastasis, *ER* estrogen receptor, *PR* progesterone receptor; HER-2: human epidermal growth factor receptor-2

### Sequencing data quality control

For the WES, the average clean reads, clean data, mapped ratio, duplicated ratio, on-target ratio, coverage, Q20% and Q30% of these 20 pairs of samples were 98,960,183.63 (72666253–156,872,275), 14.84Gb (10.90–23.53Gb), 99.98% (99.97–99.99%), 20.86% (18.03–22.78%), 71.59% (62.85–81.02%), 96.06 (72.78–144.39), 97.70% (96.72–98.25%) and 93.65% (91.27–95.00%), respectively (supplementary Table 1).

**Fig. 1:**
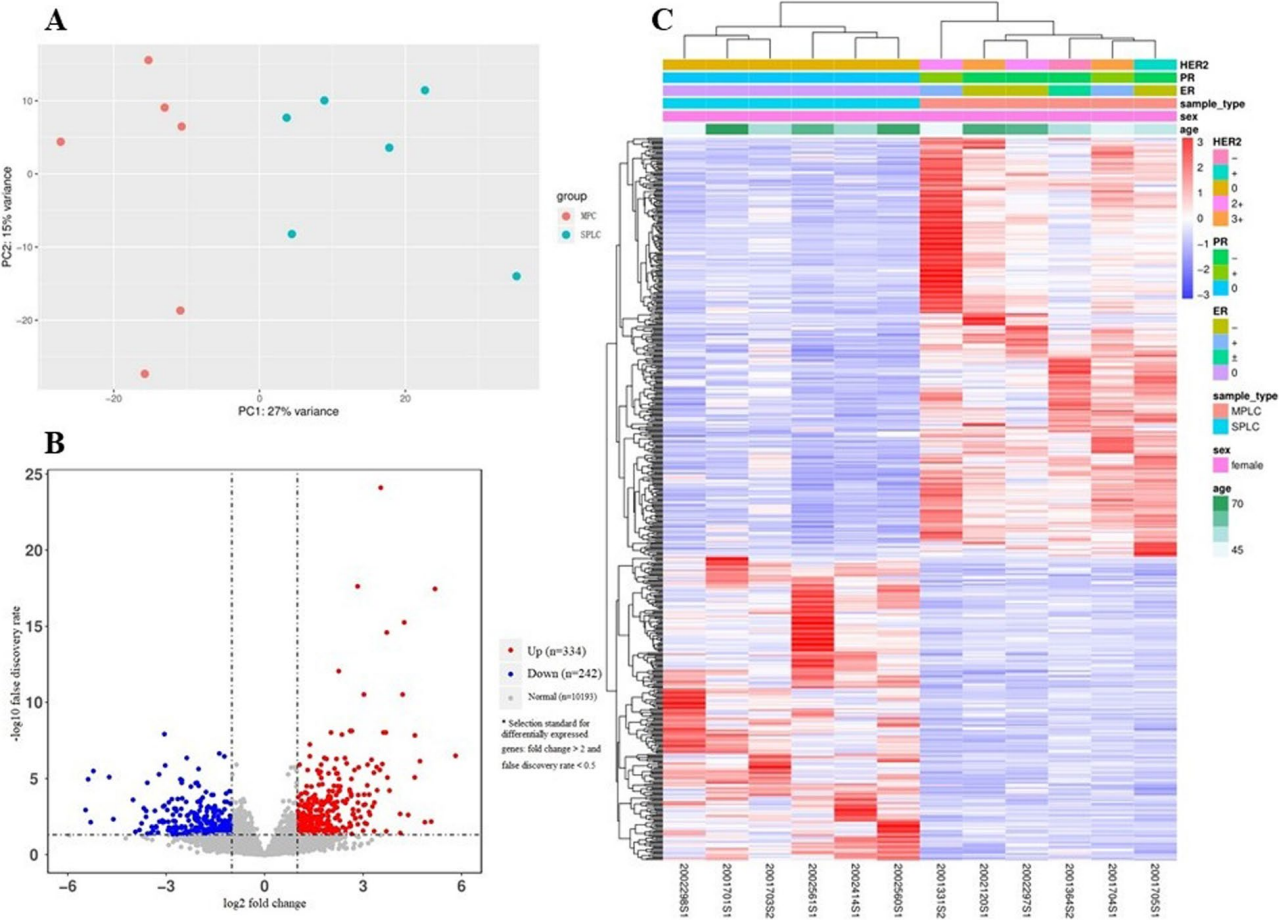
Results of principle component analysis (**A**), volcano plot (**B**) and clustering plot (C) of differentially expressed genes of 12 patients

For the RNA-seq, 18 patients including ten MPC patients and eight SPLA patients were enrolled (excluding the YY and YJH). The average clean reads, total mapped ratio, uniquely mapped ratio, duplicated ratio, Q20%, Q30% and OD260/280 were 49,943,286.89 (11834911–73,612,182), 97.97% (94.54–98.49%), 87.94% (81.39–89.20%), 12.06% (10.80–18.61%), 98.29% (97.88–98.47%), 94.91% (93.99–95.37%) and 1.915 (1.75–1.98), respectively (supplementary Table 2). However, the results of PCA and differentially expressed gene clustering showed that the clustering of four patients in the MPC group (ID: 2000808, 2,001,589, 2,002,259 and 2,001,347) and two patients in the SPLC group (ID: 2002388 and 2,001,631) was obviously poor (supplementary Fig. 1A and B). Thus, the six patients were excluded in further analysis. The results of PCA, differentially expressed genes and clustering of the remaining 12 patients were presented in the Fig. 1A, B and C, respectively.

### Results of the WES

#### SNV/InDel analysis

**Fig. 2:**
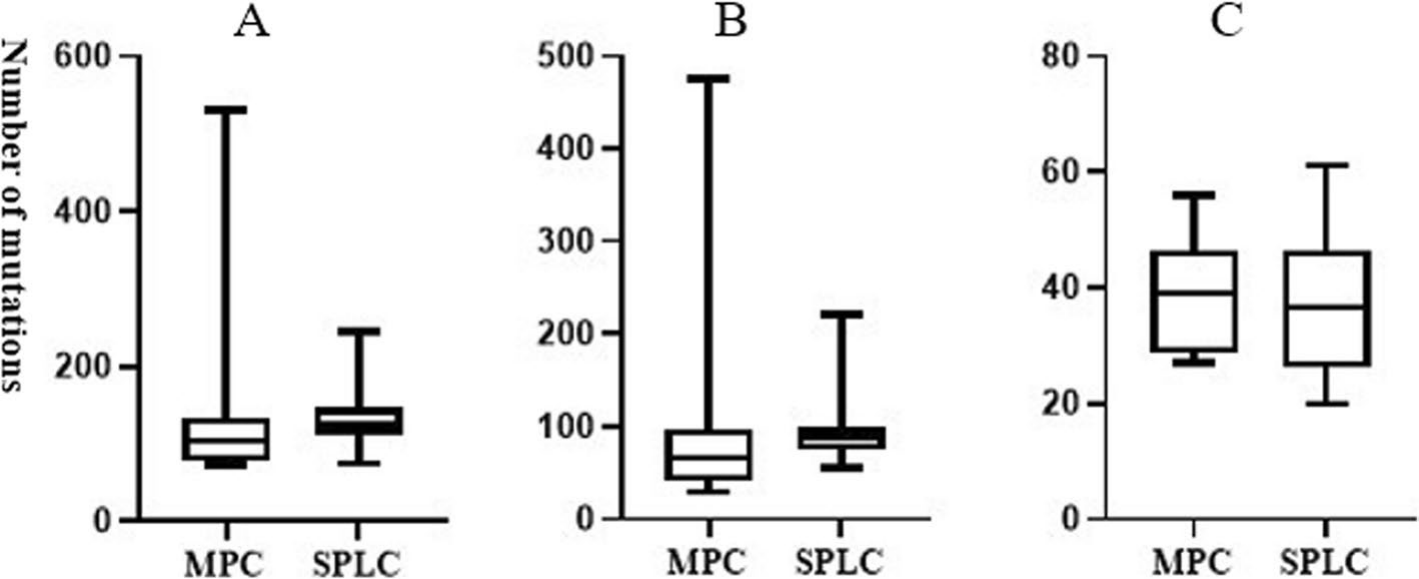
Distribution of the numbers of short mutations (**A**), SNV (**B**) and InDel (**C**) in the two groups

According to the WES, 98631 short mutations (SNV/InDel) were detected. To ensure the reliability of the results, only 2803 short mutations with alt_depth ≥ 6 were included in further analysis. In detail, 2045 SNV mutations including 700 synonymous mutations, 1195 nonsynonymous mutations, 69 splicing mutations, 38 stopgain mutations, 3 stoploss mutations and 40 unknown mutations and 758 InDel mutations including 4 non-frameshift insertions, 661 non-frameshift missings, 17 frameshift insertions and 76 frameshift missings were detected and further analyzed (Table 2). According to the Mann-Whitney test, no statistically significant differences between the two groups in short mutations (*P* = 0.112), SNV (*P* = 0.089) or InDel (*P* = 0.622) were observed (Fig. 2).

**Table 2:** Detailed information about the SNV/InDel of these twenty patients

ID	Synonymous mutation	Nonsynonymous mutation	Splicing	Stopgain	Stoploss	Unknown	Non-frameshift insertion	Non-frameshift missing	Frameshift insertion	Frameshift missing
2,000,808	180	267	10	5	0	13	2	45	1	8
2,001,331	25	38	2	0	0	4	0	23	0	4
2,001,347	18	52	5	1	0	0	1	44	0	1
2,001,364	23	37	2	0	1	2	0	42	0	4
2,001,589	46	79	2	6	1	0	0	31	0	5
2,001,704	11	14	1	2	0	2	0	36	2	4
2,001,705	16	22	2	0	0	1	0	30	0	3
2,002,120	16	31	2	0	0	1	0	25	3	1
2,002,259	28	54	1	0	0	2	0	24	1	2
2,002,297	15	23	1	2	0	0	0	45	0	3
2,001,631	24	38	4	4	0	0	0	40	1	5
2,001,701	25	43	4	4	0	0	0	25	0	2
2,001,703	29	51	3	2	1	3	0	30	1	5
2,002,031	31	62	2	1	0	2	0	36	0	4
2,002,191	37	51	1	0	0	3	0	29	0	0
2,002,298	81	128	6	3	0	3	0	20	3	1
2,002,388	19	48	9	1	0	1	0	42	0	6
2,002,414	17	34	4	0	0	0	0	19	0	1
2,002,560	24	57	6	1	0	0	0	26	2	9
2,002,561	35	66	2	6	0	3	1	49	3	8

#### SCNV analysis

**Fig. 3:**
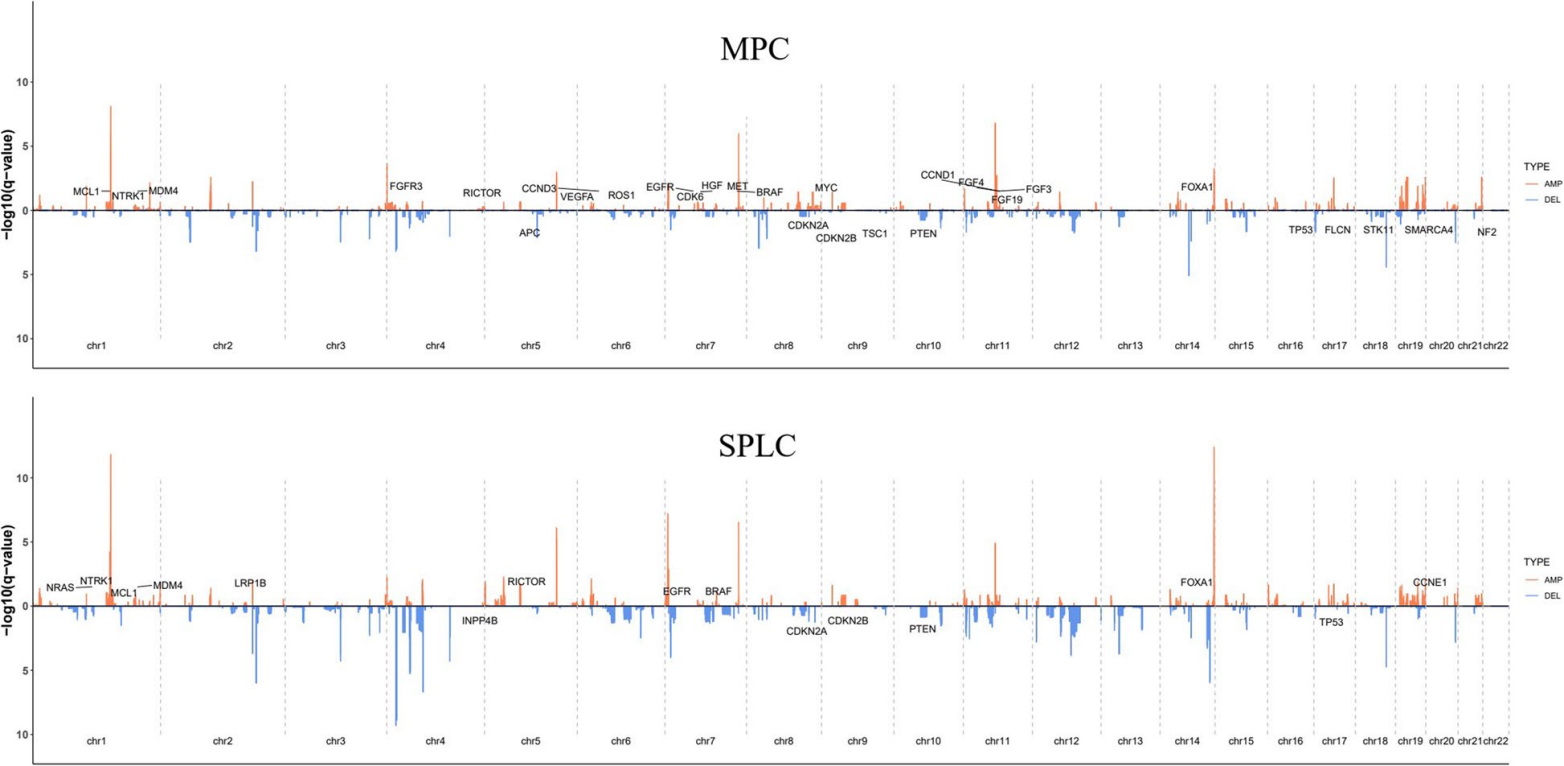
Results of the somatic copy number variation analysis

The results of the SCNV analysis were presented in the Fig. 3. No obvious differences in the gene amplification (*P* = 0.372) or deletion (*P* = 0.804) between the two groups were observed, but two patients (ID: 2000808 and 2,001,589) in the MPC group showed significant SCNV (supplementary Table 3, supplementary Fig. 2A and B).

#### Mutation spectrum analysis

**Fig. 4:**
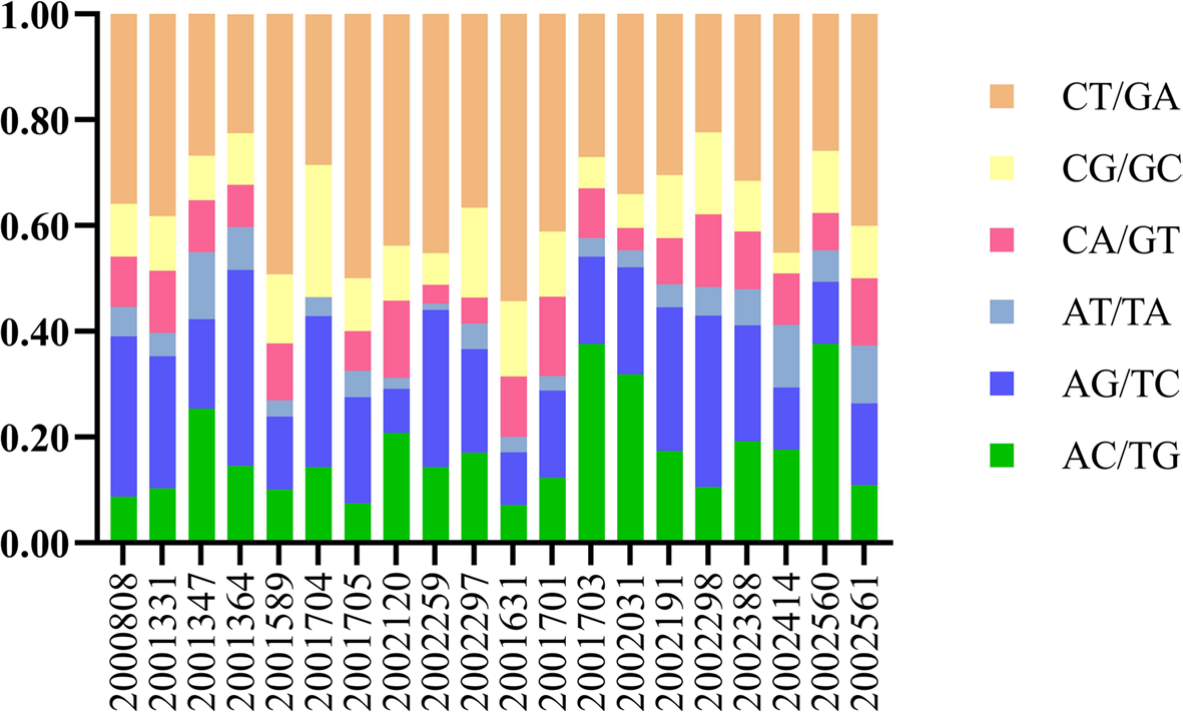
Proportions of major base substitutions

The occurrence frequencies of six main base substitutions were estimated (Fig. 4) and the results indicated that the proportion of C > T/G > A was the highest in both groups (MPC: 37%; SPLC: 32%) (supplementary Fig. 3).

**Fig. 5:**
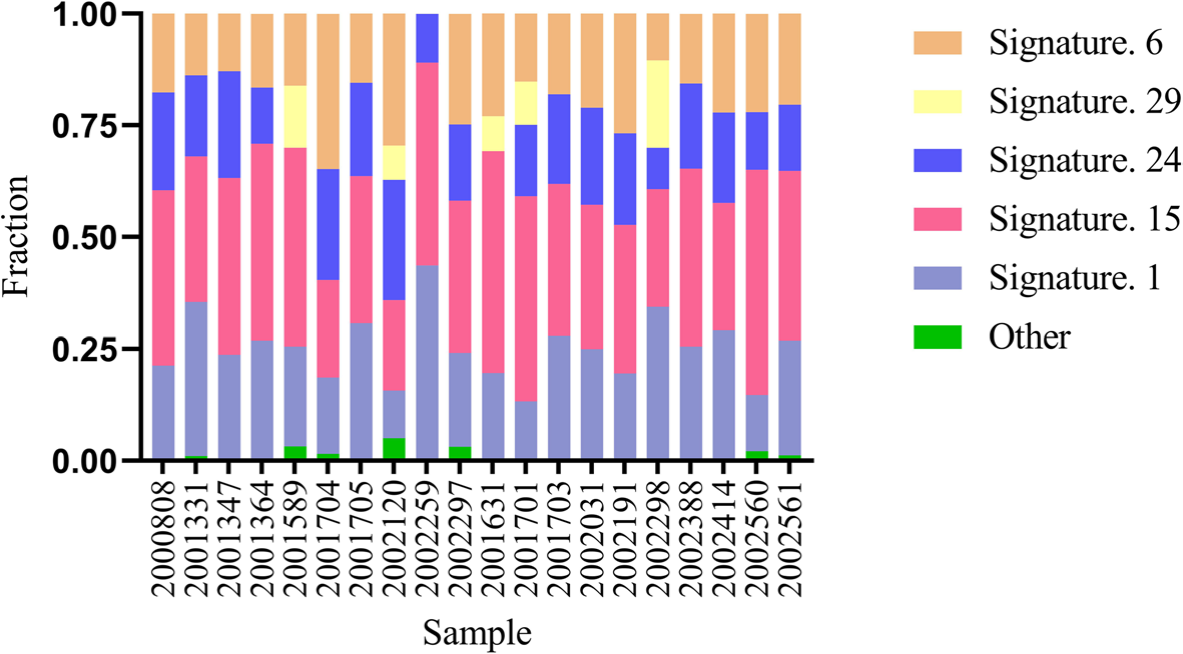
Results of mutation feature analysis

The deconstructSigs analysis were conducted to calculate the weight of the 30 tumor mutation signatures of each sample according to the COSMIC database. The results indicated that the Signature 1 (20/20, median weight: 0.243), 15 (20/20, median weight: 0.360), 6 (19/20, median weight: 0.181), 24 (18/20, median weight: 0.196) and 29 (5/20, median weight: 0.097) were common mutation signatures (Fig. 5). Besides, no significant differences in the weight of these signatures between the two groups were found (Signature 1: *P* = 0.604; 15: *P* = 0.546; 6: *P* = 0.811; 24: *P* = 0.289; 29: *P* = 0.787).

#### MSI and TMB analyses

For the MSI, all patients were defined as MSS (MSI < 20) and no significant difference in the MSI between the MPC and SPLC patients was observed (3.86 ± 3.61 vs 2.66 ± 1.95, *P* = 0.367) (supplementary Fig. 4A).

Two patients in the MPC group were defined as TMB-H (ID 2000808: 58) and TMB-M (ID 2001589: 32) separately, and two patients in the SPLC group were defined as TMB-M (ID 2002298: 25; ID 2002561: 31). No obvious differences in the TMB between the two groups were detected (13.00 ± 18.04 vs 13.70 ± 7.87, *P* = 0.912) (supplementary Fig. 4B).

#### High-frequency mutation gene analysis

**Fig. 6:**
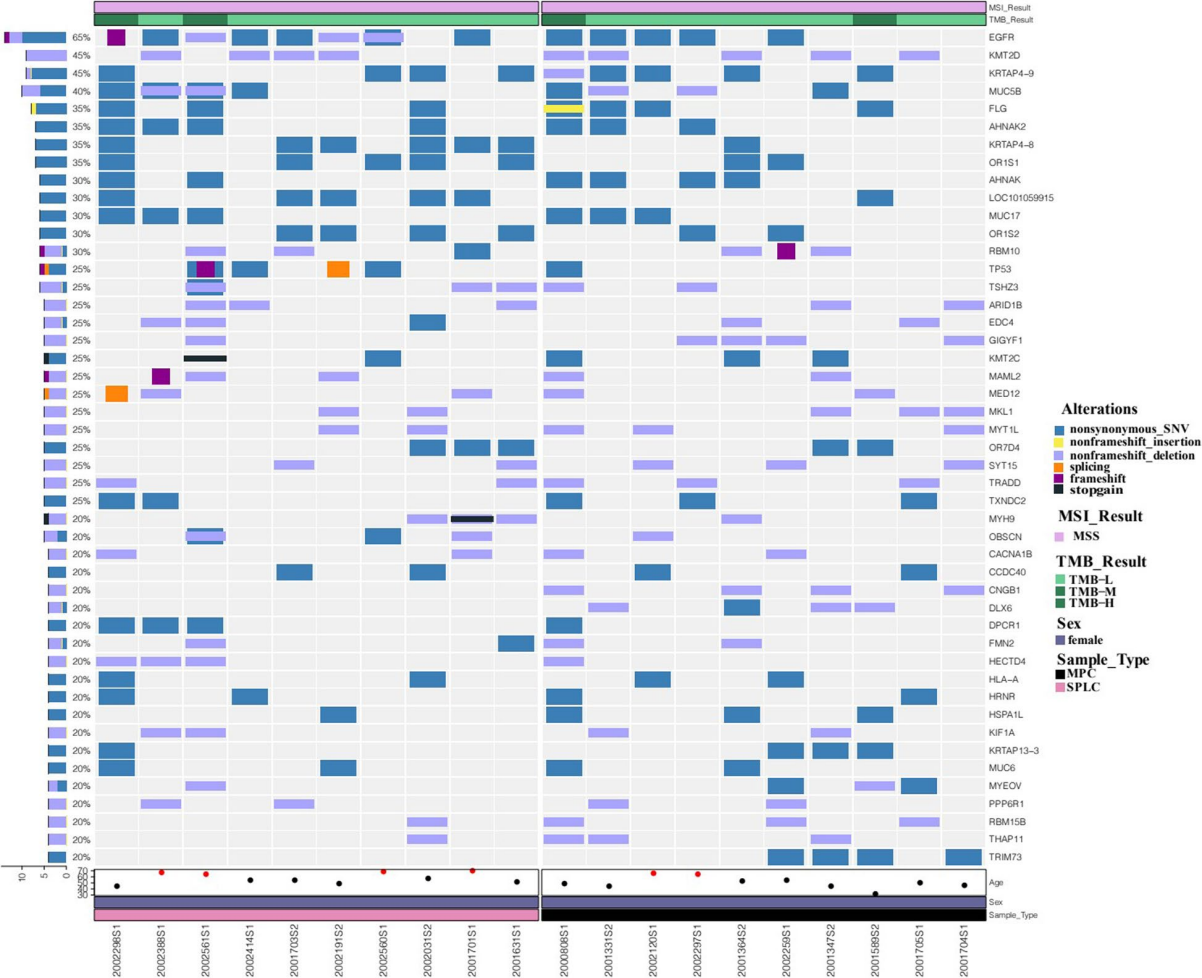
Waterfall diagram of gene mutations

The waterfall plot of high-frequency mutation genes was drawn based on above mentioned mutation information and genes that appeared in at least four samples were listed in the plot (Fig. 6). According to the waterfall plot, The TOP ten high-frequency mutation genes in this study were EGFR (65%), KMT2D (45%), KRTAP4–9 (45%), MUC5B (40%), FLG (35%), AHNAK2 (35%), KRTAP4–8 (35%), OR1S1 (35%), AHNAK (30%) and LOC101059915 (30%). There were no significant differences in the frequencies of above gene mutations between the two groups.

Interestingly, the mutations of three genes including the TRIM73, DLX6 and CNGB1 only existed in the MPC group, and the mutation frequencies of these three genes were all 40% (4/10); although the significant statistical difference was not reached (*P* = 0.087). The detailed mutation information about these three genes were presented in the Table 3. Besides, according to The Cancer Genome Atlas (TCGA) database, the mutation frequencies of TRIM73, DLX6 and CNGB1 in pulmonary tumor tissues were 0.35% (2/567), 0.94% (10/1062) and 2.82% (30/1062).

**Table 3:** Mutation information of TRIM73, DLX6 and CNGB1

Gene name	ID	Chromosome	REF	ALT	ExonicFunc	AAChange	cytoBand
TRIM73	HSQ	7	T	C	nonsyn-SNV	NM_198924;c.23 T > C;p.L8P	7q11.23
TRIM73	LHY	7	T	C	nonsyn-SNV	NM_198924;c.23 T > C;p.L8P	7q11.23
TRIM73	ZXL	7	T	C	nonsyn-SNV	NM_198924;c.23 T > C;p.L8P	7q11.23
TRIM73	ZXH	7	T	C	nonsyn-SNV	NM_198924;c.23 T > C;p.L8P	7q11.23
DLX6	HP	7	C	T	nonsyn-SNV	NM_005222;c.146C > T;p.P49L	7q21.3
DLX6	HYH	7	GGCA	G	non-shift_del	NM_005222;c.75_77del; p.Q44Pfs*250	7q21.3
DLX6	LHY	7	GGCA	G	non-shift_del	NM_005222; c.75_77del;p.Q44Pfs*250	7q21.3
DLX6	ZXH	7	GCAC	G	non-shift_del	NM_005222;c.256_258del; p.H91Qfs*203	7q21.3
CNGB1	HP	16	TTCC	T	non-shift_del	NM_001297;c.1101_1103del; p.E371Vfs*881	16q21
CNGB1	HSQ	16	TTCC	T	non-shift_del	NM_001297;c.1101_1103del; p.E371Vfs*881	16q21
CNGB1	LHY	16	TTCC	T	non-shift_del	NM_001297;c.1101_1103del; p.E371Vfs*881	16q21
CNGB1	QY	16	TTCC	T	non-shift_del	NM_001297;c.1101_1103del; p.E371Vfs*881	16q21

### Results of the RNA-seq

#### The hallmark gene sets (h.all.v7.2.Symbols.Gmt) defined as predefined gene sets

A total of 10,769 genes were involved during the GSEA. Three hundred and thirty-four were highly expressed in the MPC group and 242 genes were highly expressed in the SPLC group. Among the 50 gene sets of hallmark gene sets, only one gene set called TNFA_SIGNALING_VIA_NFKB was enriched and relatively down-regulated in the MPC group (supplementary Fig. 5 and supplementary Table 4). However, the significant statistical difference was not reached (P.adjust>0.05).

#### The GO gene sets (c5.All.v7.2.Symbols.Gmt) applied as predefined gene sets

Among the 10,271 functional gene sets, 365 enriched and up-regulated gene sets in the MPC group were detected based on the standards of |NES| ≥ 1, FDR q-value < 0.05 and NOM *p*-value < 0.05, including 294 biological process (BP) related, 18 molecular function (MF) related and 53 cell component (CC) related gene sets (supplementary Table 5). Besides, 28 significantly down-regulated gene sets, including 17 BP related, 2 MF related and 9 CC related gene sets, were screened out (supplementary Table 6). Nearly half (192/393) of the enriched gene sets were significantly associated with tumor occurrence, invasion, metastasis or drug response.

**Fig. 7:**
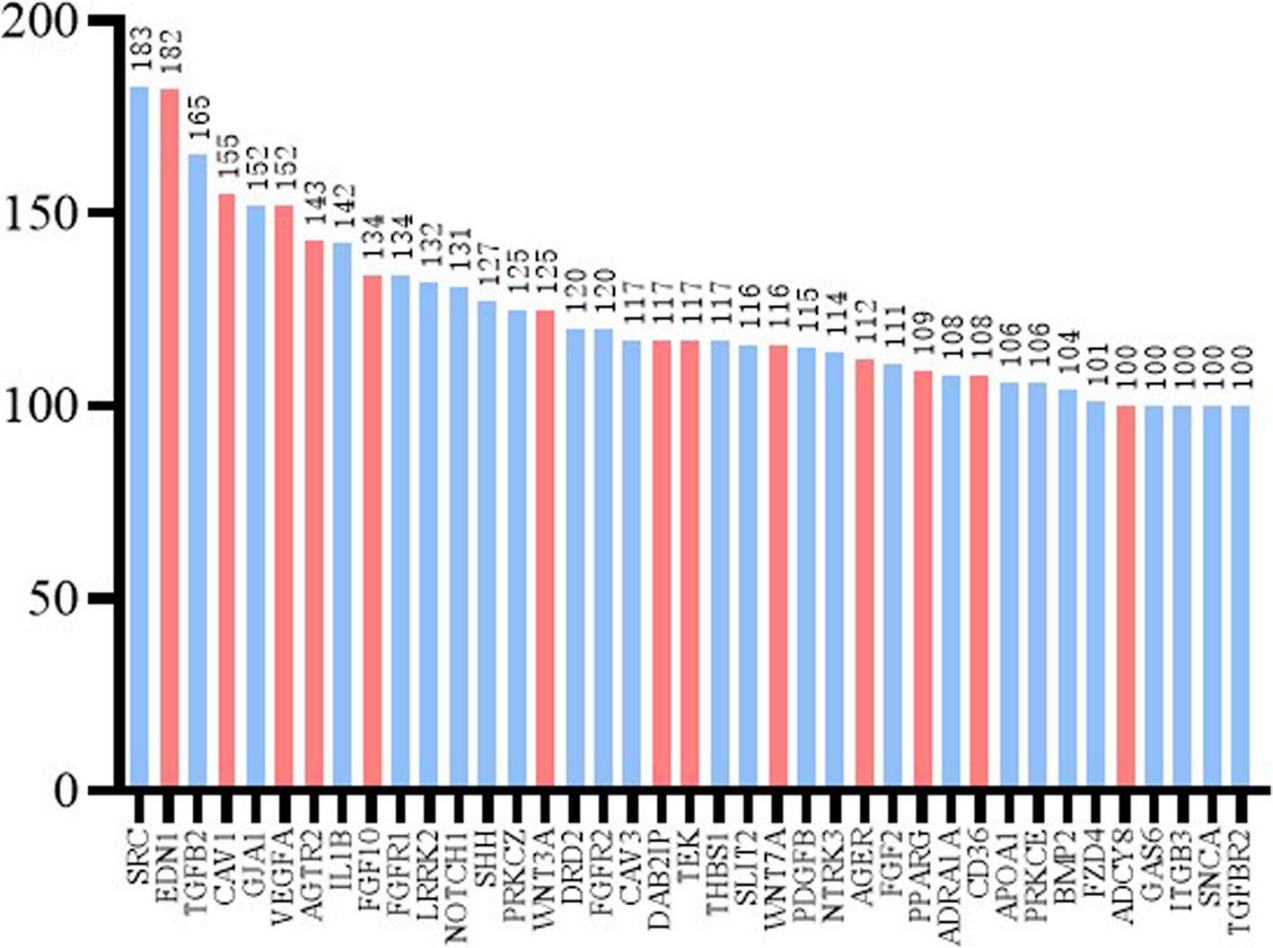
High-frequency genes in the all GO analysis

The leading edge analysis based on above significantly up-regulated or down-regulated gene sets was further conducted to identify high-frequency genes. Thirty-nine high-frequency genes that appeared at least 100 enriched gene sets were identified (Fig. 7A) and the TOP10 genes were SRC (183/365), EDN1 (182/365), TGFB2 (165/365), CAV1 (155/365), GJA1 (152/365), VEGFA (152/365), AGTR2(143/365), IL1B (142/365), FGF10 (134/365) and FGFR1 (134/365). Among these 39 high-frequency genes, the expression levels of 13 genes in the MPC group increased significantly, including the EDN1, CAV1, VEGFA, AGTR2, FGF10, WNT3A, DAB2IP, TEK, WNT7A, AGER, PPARG, CD36 and ADCY8, and the detailed information about these 13 genes were presented in supplementary Table 7. Furthermore, except for ADCY8, the up-expression of above genes were all related to tumor development, metastasis, invasion, sensitivity to radiotherapy and chemotherapy or prognosis.

#### The KEGG gene sets (c2.Cp.Kegg.v7.2.Symbols.Gmt) applied as predefined gene sets

**Fig. 8:**
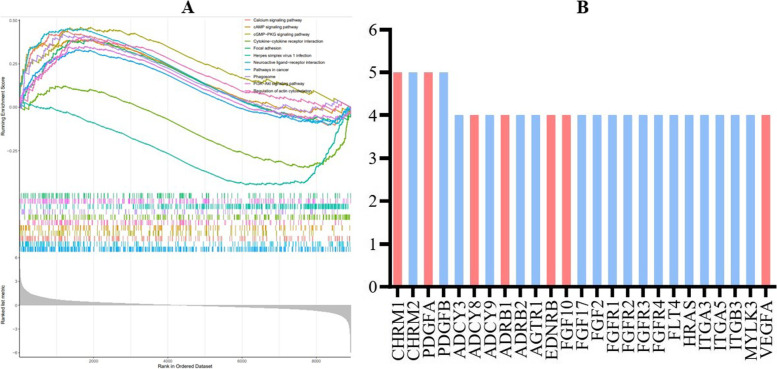
Enrichment analysis results of KEGG functional gene sets (**A**) and high-frequency genes in the KEGG analysis (**B**)

Among the 186 gene sets, nine enriched and up-regulated and two down-regulated pathways were screened out based on the standards of |NES| ≥ 1, FDR q-value < 0.05 and NOM *p*-value < 0.05 (Table 4, Fig. 8A). Most of them (8/11) were related with tumor development, invasion and metastasis.

**Table 4:** Significantly up-regulated or down-regulated gene sets in the KEGG analysis

Name	Size	ES	NES	*P* value	*P*.adjust value	FDR *q*-value
Pathways in cancer	422	0.331	1.466	0.001	0.024	0.017
Neuroactive ligand-receptor interaction	185	0.453	1.846	0.001	0.024	0.017
Calcium signaling pathway	177	0.439	1.777	0.001	0.024	0.017
Phagosome	113	0.430	1.622	0.002	0.024	0.017
cGMP-PKG signaling pathway	122	0.460	1.752	0.002	0.024	0.017
Regulation of actin cytoskeleton	150	0.400	1.570	0.003	0.038	0.026
Herpes simplex virus 1 infection	329	−0.445	−2.069	0.004	0.038	0.026
PI3K-Akt signaling pathway	246	0.350	1.469	0.004	0.038	0.027
Focal adhesion	157	0.391	1.550	0.005	0.038	0.027
cAMP signaling pathway	168	0.390	1.558	0.006	0.044	0.031
Cytokine-cytokine receptor interaction	210	−0.347	−1.532	0.006	0.044	0.031

*ES* enrichment score, *NES* normalized enrichment score, *FDR* false discovery rate

Similarly, the leading edge analysis was also performed and 25 high-frequency genes that appeared at least four enriched gene set were identified (Fig. 8B). Among these 25 genes, the expression levels of seven genes in the MPC group increased significantly, including the CHRM1 (5/11), PDGFA (5/11), ADCY8 (4/11), ADRB1 (4/11), EDNRB (4/11), FGF10 (4/11) and VEGFA (4/11). Interestingly, except for ADCY8, the up-expression of above genes were all significantly related to tumor development, metastasis, invasion, sensitivity to radiotherapy and chemotherapy or prognosis.

Therefore, according to the results of all GO and KEGG analyses, FGF10 and VEGFA might play an important role in the development of second primary lung cancer in breast cancer patients.

## Discussion

According to the results of our research, the occurrence of second primary lung adenocarcinoma may be related to the cytokine-cytokine receptor action, autophagy, PI3L-Akt, cAMP and calcium ion signaling pathways. Furthermore, the mutations of TRIM73, DLX6 and CNGB1 and high expression of FGF10 and VEGFA might play an important role in the development of lung adenocarcinoma in breast cancer patients. Thus, these pathways and genes may be important targets for us to reduce the incidence of lung adenocarcinoma after treatment of breast cancer. However, more in-depth investigations focusing on these targets are needed to verify above conjectures.

The result of high-frequency gene analysis demonstrated that the mutations of three genes including the TRIM73, DLX6 and CNGB1 only existed in the MPC group, and the mutation frequencies of these three genes were all 40%. However, the mutation frequencies of these three genes in the TCGA database were reported to be 0.35, 0.94 and 2.82%, respectively. Thus, TRIM73, DLX6 and CNGB1 may be relatively characteristic genes in pulmonary adenocarcinoma patients with previous breast cancer. Tripartite motif containing 73 (TRIM73) is a member of the TRIM family and is also called Tripartite motif-containing protein 50B (TRIM50B). It has been reported that the TRIM73 might act as an E3 ubiquitin ligase [8]. However, literatures about the TRIM73 are still rare, especially its biological function in tumors. Up to now, only Li et al. reported that the supermethylation of TRIM73 in plasma could be applied as an important indicator for the early diagnosis of pancreatic cancer [9]. Actually, the clinical values of TRIM family genes in tumorigenesis, development and prognosis have been manifested by a number of relevant studies and most of members of the TRIM family play a role in tumors as proto-oncogenes or tumor-promoting genes [10–15]. For example, the TRIM50 could enhance the proliferation, cloning, invasion and migration abilities of oral squamous cell carcinoma by reducing the expression level of retinoblastoma tumor suppressor protein (Rb) [16]. Furthermore, the TRIM47 gene mutation significantly affect the prognosis of liver carcinoma patients (*P* = 0.014) and patients with TRIM47 gene mutations are more likely to have poorer overall survival (OS), which might be caused by the ability of TRIM47 to promote the proliferation of liver cancer cells [17]. Besides, the overexpression of TRIM35 gene could obviously improve the proliferation, migration and invasion ability of lung cancer cells [18]. Therefore, it is crucial to further explore the specific mechanisms and clinical values of TRIM73 gene mutation in lung adenocarcinoma patients with previous breast cancer.

According to previous literatures, the distal-less homeobox 6 (DLX6) gene, a member of DLX family, plays an important role in the development of craniofacial structure, inner ear, limbs and brain [19]. Some scholars have manifested that DLX6 is mainly regulated by a new type of upstream transcribed non-coding RNA (EVF-1) and p63 and the abnormal expression of this gene may be related to the occurrence of Split Hand-Foot Malformation (SHFM) and estrogenic Ectodactyly-Ectodermal dysplasia-Cleft lip (EEC) [20, 21]. Few studies explore the association of DLX6 with cancers up to now. Only Liang et al. found that DLX6 was highly expressed in oral squamous cell carcinoma and could promote cell proliferation and inhibit cell apoptosis, which might be regulated by the Epidermal Growth Factor Receptor-Cyclin D1 (EGFR-CCND1) pathway [19]. The main role of cyclic nucleotide-gated channel subunit beta 1 (CNGB1) gene is to encode the β subunit of the rod-shaped photoreceptor cyclic guanosine monophosphate (cGMP)-gated cation channel [22, 23]. Therefore, the mutation of this gene is mainly associated with the occurrence of retinitis pigmentosa and degeneration and the mutation types include the p. (L849Afs*3), p. (L129WfsTer148), p. (A1048fs*13), etc. [22, 23]. However, the CNGB1 mutations of four patients in this study were all c.1101_1103del, p.(E371Vfs*881) (non-frameshift missing) and no such mutation has been reported up to now. Thus, the biological significance of DLX6 and CNGB1 gene mutations in lung adenocarcinoma patients with breast cancer still needs to be further investigated.

In this study, the KEGG gene sets (c2.cp.kegg.v7.2.symbols.gmt) was applied as predefined gene set for GSEA analysis and nine significantly up-regulated and two down-regulated pathways were found, including eight tumor-related pathways (Pathways in cancer, Calcium signaling pathway, Phagosome, Regulation of actin.

cytoskeleton, PI3K-Akt signaling pathway, Focal adhesion, cAMP signaling pathway, Cytokine-cytokine receptor interaction). Similarly, 365 significantly enriched and up-regulated gene set and 28 down-regulated gene sets were screened out after the all GO analysis. Besides, the leading edge analyses were performed based on the enriched gene sets identified by all GO and KEGG analyses and 13 high-frequency genes (EDN1, CAV1, VEGFA, AGTR2, FGF10, WNT3A, DAB2IP, TEK, WNT7A, AGER, PPARG, CD36, ADCY8) and seven high-frequency genes (CHRM1, PDGFA, ADCY8, ADRB1, EDNRB, FGF10, VEGFA), respectively. Based on above findings and literature review, we mainly focused on the FGF10 and VEGFA in further investigation.

Fibroblast growth factor 10 (FGF10) mainly activates fibroblast growth factor receptor 2 (FGFR2) (FGFR2b) AND FGFR1 (FGFR1b) on the surface of epithelial derived cells and signals in the form of paracrine. It has been verified that FGF10 is essential for the development of the brain, lungs and limbs and help wound healing and tissue repair by promoting cell migration and proliferation [24, 25]. Therefore, when above-mentioned functions of FGF10 are disturbed and abnormal signals are transmitted through FGFR2b/FGFR1b, it may lead to the tumorigenesis [26]. It has been reported that the FGF10-FGFR2b signaling pathway plays a central role in the development of the breast and FGF10 is not expressed in normal human breast ductal epithelial cells [27, 28]. However, the significant increase in the transcription level of FGF10 was observed in about 10% of breast cancer patients [29]. Besides, variation near the FGF10 gene locus are genetic risk factors for the susceptibility of breast cancer and FGF10 is an oncogene of breast cancer [30]. FGF10 plays a role in promoting the occurrence and development of breast cancer mainly due to the following causes. First, activated FGFR2 could significantly inhibit the activity of ER modulators and reduce the sensitivity of breast cancer patients to anti-estrogen therapy [31, 32]. Therefore, the FGF10-FGFR2 signaling pathway might be a new target to reduce resistance to the estrogen therapy [31, 32]. Second, activated FGFR2 also plays a role in promoting the receptor recycling, which leads to the migration of breast tumors [26]. Third, FGFR10 could also promote the high expression of some genes that regulate cell migration and invasion after it is activated by FGF10 [33]. This process is affected by the activity of granzyme B, but at the same time FGF10 shows a positive regulatory effect on granzyme B, which ultimately leads to the enhancement of breast tumor invasion and migration ability [33]. Similarly, FGF10 has also been reported to play an essential role in the occurrence and progression of lung cancer and its overexpression in respiratory epithelial cells can easily lead to the occurrence of multifocal pulmonary adenocarcinoma [34]. Previous studies have shown that FGF10 secreted by tumor-associated macrophages (TAMs) are able to boost the growth of lung cancer, but the detailed mechanisms behind this enhancement remain unclear now [35]. Furthermore, FGF10 plays an important role in several significantly up-regulated signaling pathways, including the Pathways in cancer, Calcium signaling pathway, Regulation of actin cytoskeleton and PI3K-Akt signaling pathway. Thus, above findings suggest that FGF10 may be one of the characteristic genes of lung adenocarcinoma patients with breast cancer.

Vascular endothelial growth factor-A (VEGFA) is one of the most effective promotors for angiogenesis and lymphangiogenesis. It often binds to specific receptors such as the vascular endothelial growth factor receptor 1 (VEGFR1) and VEGFR2 [36, 37]. VEGFA gene is mainly expressed in angioblasts and endothelial cells, and is related to the growth, movement and vascular permeability of endothelial cells [36, 37]. At present, a large number of studies have confirmed that VEGFA plays a vital role in tumor growth, metastasis and angiogenesis [38, 39]. VEGFA binds to its receptors to initiate multiple signal cascades, which in turn leads to the proliferation, migration and differentiation of endothelial cells [38, 39]. Some scholars have found that expression level of VEGFA in the blood vessels and cells of a variety of tumors increase exponentially, and the expression status of VEGFA is significantly associated with the disease progression and prognosis of tumor patients [40–44]. Several anti-angiogenesis targeted drugs for VEGFA and its receptor (VEGFR2) have been developed such as the bevacizumab and ramucirumab which could be used for advanced non-small cell lung cancer (NSCLC) [43, 45]. However, it is unclear whether they have certain clinical values in the treatment of breast cancer and reducing the incidence of second lung cancer of breast cancer patients. Furthermore, after comprehensive literature searching, we found that the VEGF gene polymorphism was closely associated with the occurrence, clinical therapeutic effects and prognosis for both lung cancer and breast cancer [46–52]. Thus, it is also necessary to explore the association of VEGFA gene polymorphism with the occurrence of second primary lung cancer in breast cancer patient in future relevant studies.

At the same time, we found that the serum expression levels of FGF and VEGF would be decreased by the endocrine therapy (tamoxifen) and chemotherapy after reviewing relevant literatures [53–56]. Besides, the expression of ERβ is significantly related to VEGF and patients with high ERβ expression also have higher VEGF levels [57]. However, it is still not clear about the effects of endocrine therapy and chemotherapy on the expression levels of FGF10 and VEGFA in breast cancer patients, which is also one of the important directions of our next research. Based on above information, we speculated that FGF10 and VEGFA might play an essential role in the screening and clinical intervention for second primary lung cancer of high-risk breast cancer patients, but further investigation should be conducted to verify this conjecture.

Although several genes and pathways were detected to be potentially related to the occurrence of second primary lung cancer in breast cancer patients, it is well known that the incidence of lung adenocarcinoma is closely associated with some driver gene mutations like the EGFR mutation. However, no significant difference in the driver gene mutations between the MPC and SPLA groups were found in this study, which might be related to the small sample size or other depth causes. Thus, the association between the occurrence of second primary lung cancer in breast cancer patients and mutations of lung cancer driver genes should be explored by more high-quality studies with big sample sizes.

In this study, MPLA patients were designed as the control group rather than single primary breast cancer (SPBC) patients for the following reasons. First, fresh specimens are required for the RNA-seq, but it is very difficult to obtain fresh breast tumor specimens of MPC patients. Second, according to our previous data, more than half of MPC patients (72/137) were diagnosed 5 years after the diagnosis of breast cancer, and pulmonary nodules or masses were not found in a considerable number of patients at the time of diagnosis of breast cancer [58]. Thus, SPBC patients cannot be designed as the control group in the strict sense, unless the follow-up time is long enough such as 10 years or even 20 years. Third, when SPLC patients were admitted to our hospital, we could ask for detailed medical history and perform the mammography or mammography, cephalo-thoracoabdominal enhanced CT and bone scan to ensure that the patient had no breast tumors and other malignancies. Therefore, we believe that it is more feasible and scientific to select SPLC patients as the control group.

One of the important limitations in the current study is that we are unable to confirm when the mutations of TRIM73, DLX6 and CNGB1 occurred. In other words, it is unclear whether the mutations of these three genes are characteristics of these patients or secondary changes after the treatment for breast cancer, which needs to be further verified by further researches. Besides, the overall sample size is relatively small, so we intend to include more samples for follow-up research.

## Conclusion

The occurrence of second primary lung adenocarcinoma may be related to the cytokine-cytokine receptor action, autophagy, PI3L-Akt, cAMP and calcium ion signaling pathways. Furthermore, the mutations of TRIM73, DLX6 and CNGB1 and high expression of FGF10 and VEGFA might play an important role in the development of lung adenocarcinoma in breast cancer patients. However, more in-depth investigations are needed to verify above findings.

## Supplementary Information

**Additional file 1: Supplementary Fig. 1.** Principal component analysis of 18 patients (A) and cluster analysis of differentially expressed genes in 14 patients (B).

**Additional file 2: Supplementary Fig. 2.** Results of somatic cell copy number variation analysis for patient QY (A) and ZXH (B).

**Additional file 3: Supplementary Fig. 3.** Proportions of major base substitutions in two groups.

**Additional file 4: Supplementary Fig. 4.** Results of microsatellite stability (A) and tumor mutation burden (B).

**Additional file 5: Supplementary Fig. 5.** Results of Hallmarks functional gene set enrichment analysis.

**Additional file 6.**

**Additional file 7.**

## Data Availability

The datasets presented in this study have been deposited in the GSA repository with the accession number of HRA001762 (https://bigd.big.ac.cn/gsa-human/browse/HRA001762).
