# Supplementary figures and images for: The genomic and transcriptome characteristics of lung adenocarcinoma patients with previous breast cancer
Source: BMC Cancer. 2022 Jun 6;22:618. doi: 10.1186/s12885-022-09727-6 (PMC9171992; doi:10.1186/s12885-022-09727-6)

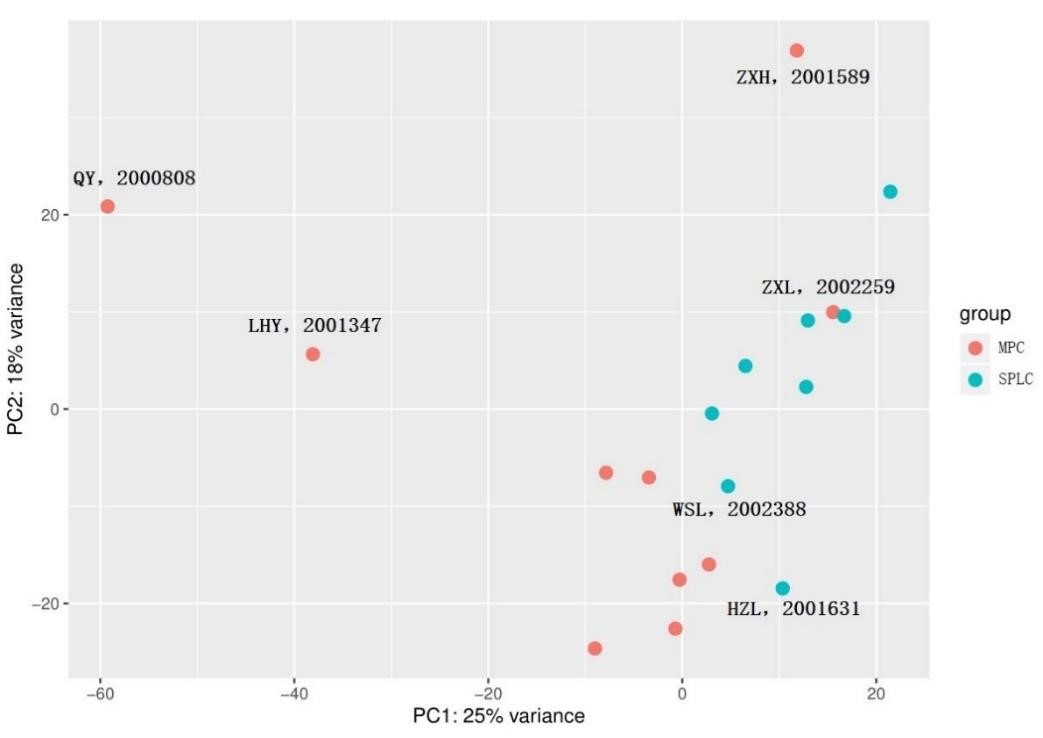

Supplement: Supplementary file 1 — Additional file 1: Supplementary Fig. 1. Principal component analysis of 18 patients (A) and cluster analysis of differentially expressed genes in 14 patients (B). [file 12885_2022_9727_MOESM1_ESM.zip › supplementary figure 1a-4a.jpg]

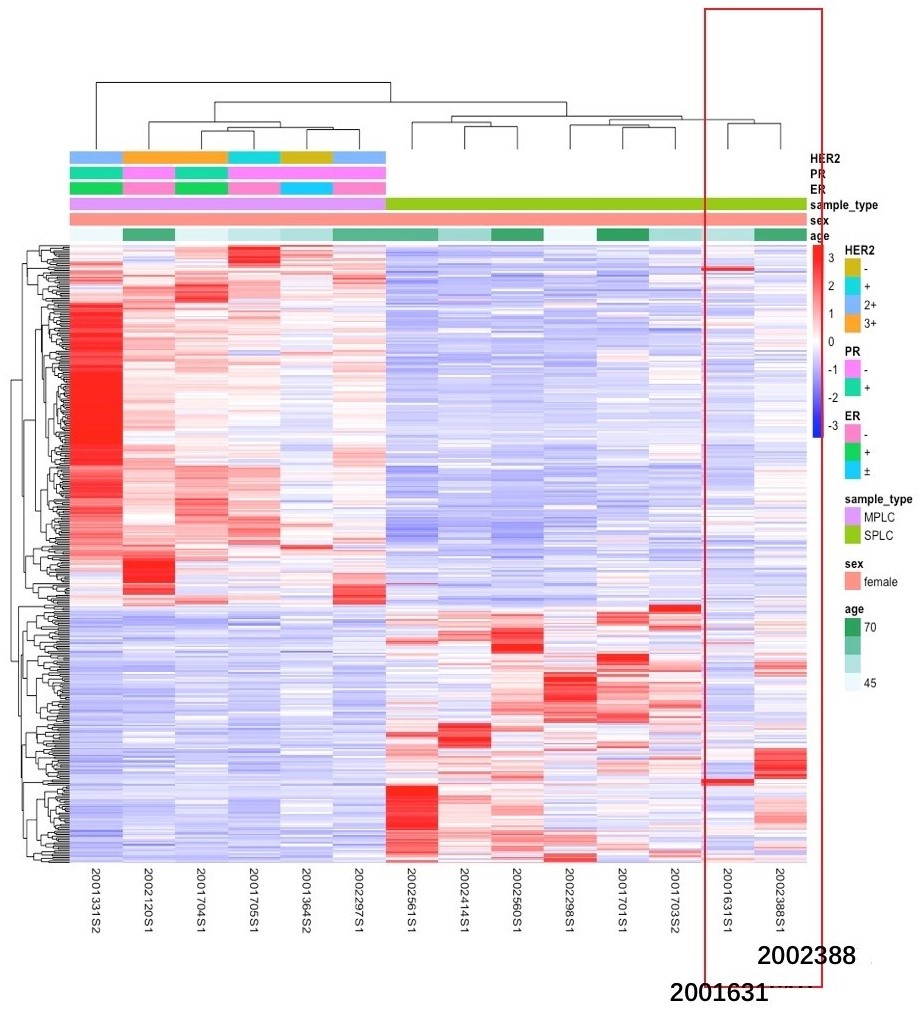

Supplement: Supplementary file 1 — Additional file 1: Supplementary Fig. 1. Principal component analysis of 18 patients (A) and cluster analysis of differentially expressed genes in 14 patients (B). [file 12885_2022_9727_MOESM1_ESM.zip › supplementary figure 1b-4b.jpg]

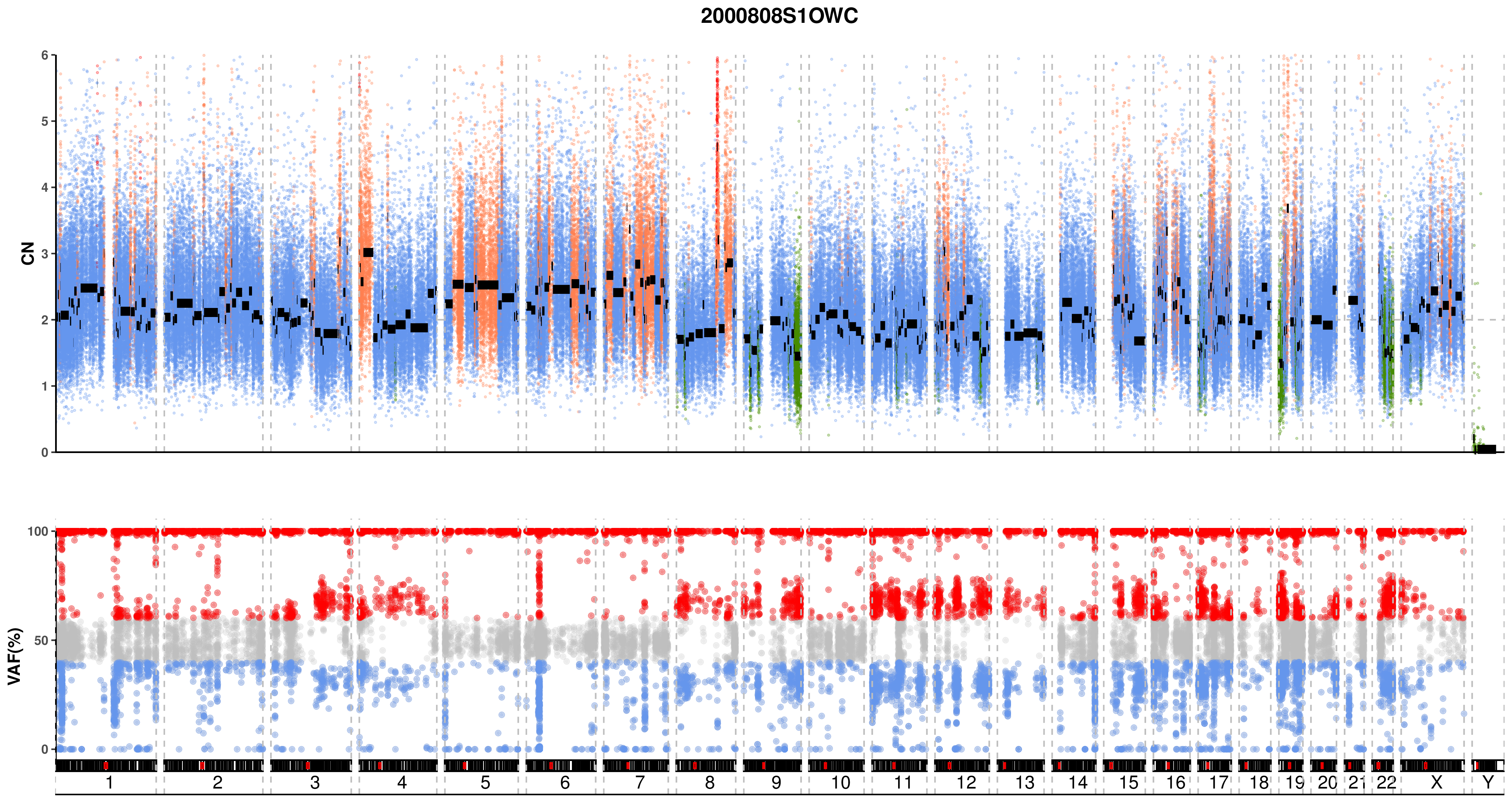

Supplement: Supplementary file 2 — Additional file 2: Supplementary Fig. 2. Results of somatic cell copy number variation analysis for patient QY (A) and ZXH (B). [file 12885_2022_9727_MOESM2_ESM.zip › supplementary figure 2A.png]

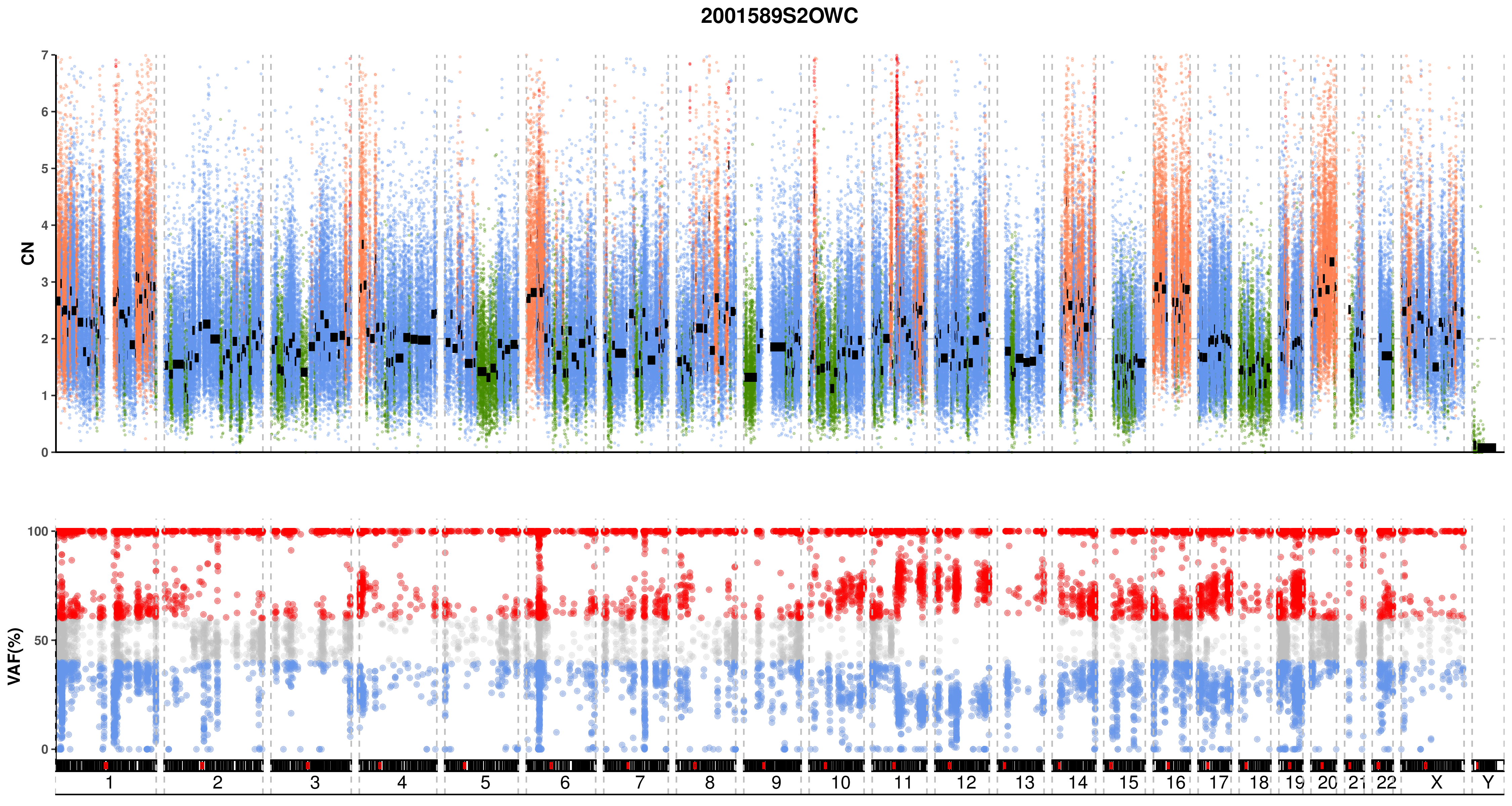

Supplement: Supplementary file 2 — Additional file 2: Supplementary Fig. 2. Results of somatic cell copy number variation analysis for patient QY (A) and ZXH (B). [file 12885_2022_9727_MOESM2_ESM.zip › supplementary figure 2B.png]

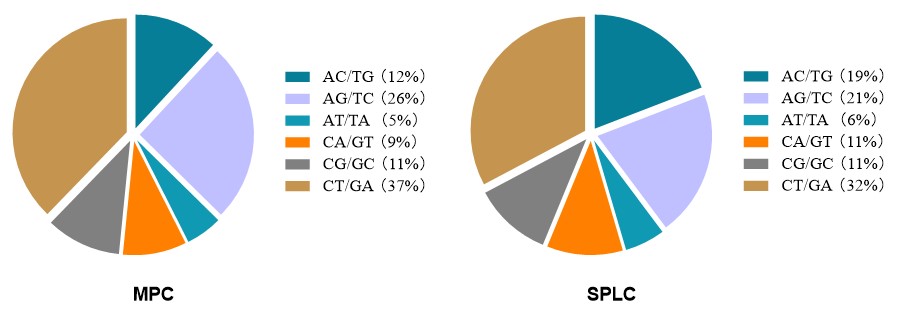

Supplement: Supplementary file 3 — Additional file 3: Supplementary Fig. 3. Proportions of major base substitutions in two groups. [file 12885_2022_9727_MOESM3_ESM.jpg]

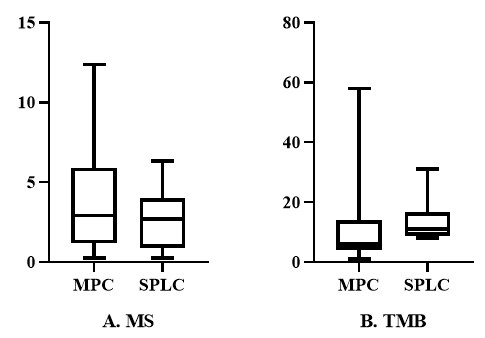

Supplement: Supplementary file 4 — Additional file 4: Supplementary Fig. 4. Results of microsatellite stability (A) and tumor mutation burden (B). [file 12885_2022_9727_MOESM4_ESM.jpg]

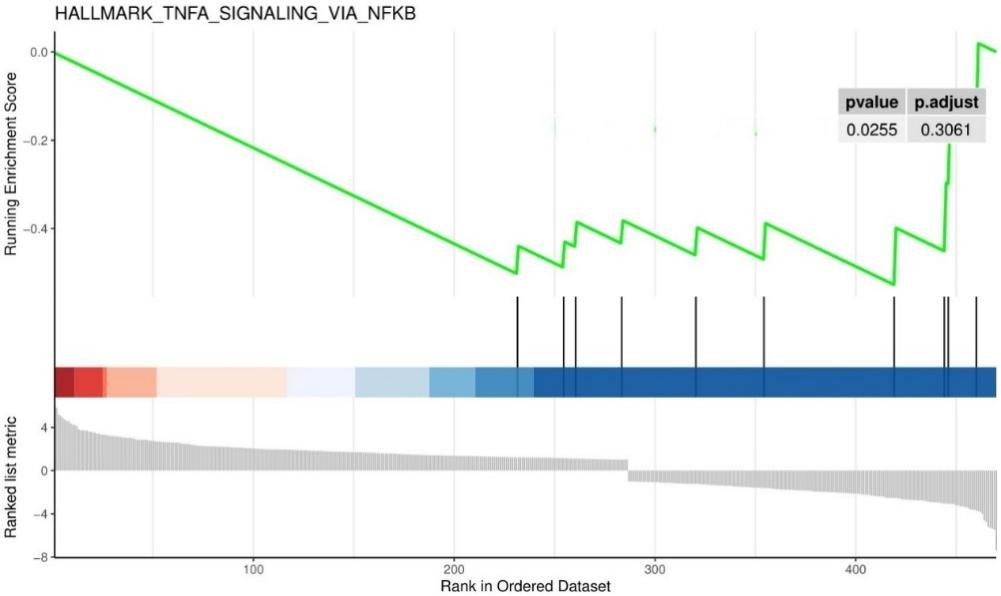

Supplement: Supplementary file 5 — Additional file 5: Supplementary Fig. 5. Results of Hallmarks functional gene set enrichment analysis. [file 12885_2022_9727_MOESM5_ESM.jpg]
