# Supplementary material for: The genomic and transcriptome characteristics of lung adenocarcinoma patients with previous breast cancer
Source: BMC Cancer. 2022 Jun 6;22:618. doi: 10.1186/s12885-022-09727-6 (PMC9171992; doi:10.1186/s12885-022-09727-6)
Supplement: Supplementary file 6 — Additional file 6. [file 12885_2022_9727_MOESM6_ESM.docx]

**DNA/RNA co-extraction using the AllPrep DNA/RNA Mini Kit:**

a. tissue lysis and homogenization;

b. adding 20ul of Proteinase K;

c. using conventional rotor-stator homogenizer to mash and homogenize tissues and adding the right amount of Buffer RLT Plus; the general reference standard for the specific usage of Buffer RLT Plus is:

| Tissue volume | Buffer RLT Plus usage |
| --- | --- |
| ＜20mg | 350ul or 600ul |
| 20-30mg | 600ul |

d. centrifuge the lysate at maximum speed for 3 minutes;

e. moving the AllPrep DNA spin column to a new 2ml collection tube and storing it at 4℃ or room temperature (15-25℃) to facilitate DNA purification in the subsequent process;

f. using a micropipette to accurately estimate the volume of the filtrate, generally 600ul or 350ul, and then adding the same volume of 70% ethanol;

g. transferring the sample (up to 700ul) to the RNeasy spin column placed in 2ml collection tube, including the formed precipitate; then centrifuging at a speed of ≥8000xg(≥10000rpm) for 15 seconds and discarding the filter liquid;

h. adding 700ul buffer RW1 to the RNeasy spin column carefully and centrifuging at a speed of ≥8000xg(≥10000rpm) for 15 seconds;

i. adding 500ul buffer RPE to the RNeasy spin column carefully and centrifuging at a speed of ≥8000xg(≥10000rpm) for 15 seconds; (twice)

j. moving the RNeasy spin column to a new 2ml collection tube, centrifuging at full speed for 1 minute and discarding the old collection tube with residual filtrate;

k. moving the RNeasy spin column to a new 1.5ml collection tube, adding 30-50ul RNase-free water to the spin column membrane and centrifuging at a speed of ≥8000xg(≥10000rpm) for a minute; (twice)

l. adding 500ul of buffer AW1 to the AllPrep DNA spin column in step e and centrifuging at a speed of ≥8000xg(≥10000rpm) for 15 seconds;

m. adding 500ul buffer AW2 to the AllPrep DNA spin column and centrifuging at full speed for 2 minutes;

n. moving the AllPrep DNA spin column to a new 1.5ml collection tube, adding 100ul EB buffer to the spin column membrane, incubating at room temperature (15-25℃) for 1 minute and then centrifuging at a speed of ≥8000xg(≥10000rpm) for a minute; (twice).

**The specific equipment used for the WES were as follows:**

| Device name | Equipment manufacturer |
| --- | --- |
| AMPure XP | Beckman Coulter, America |
| Illumina HiseqX TEN | Illumina, America |
| NanoDrop 2000 | Thermo, America |
| Covaris disruptor | Covaris, America |
| cBot | Illumina, America |
| Barnstead | Millipore, America |
| Labchip | Agilent, America |
| Qubit2.0 | Life, America |
| q225 real-time qPCR | Kubo, America |

**RNA-seq**

**RNA quantification and detection:**

a. using the 1% agarose gel electrophoresis to detect RNA degradation and contamination;

b. using the Qubit® RNA detection kit to measure RNA concentration;

c. using the Agilent 2100RNA Nano 6000 Assay kit to detect RNA integrity;

d. using the NanoPhotometer® spectrophotometer to detect RNA concentration.

**Library construction and quality control:**

a. purifying mRNA using poly-T oligomer magnetic beads;

b. adding divalent cations to NEBNext first-strand synthesis reaction buffer to make it cleave at high temperature;

c. using M-MuLV reverse transcriptase and random oligonucleotides as primers to synthesize first-strand cDNA;

d. using ribonuclease H and DNA polymerase 1 to synthesize second-strand cDNA and converting the remaining catenary into blunt ends by polymerase/exonuclease activity;

e. after the 3’ end of the cDNA fragment is adenylated, connect the NEBNext adaptor;

f. using the AMPure-XP system to screen cDNA fragments of about 200 bp, ligating the cDNA at 37°C for 15 minutes, placing it at 95°C for 5 minutes, and then performing PCR amplification;

g. purifying the PCR products (AMPure XP beads), and then using the Agilent Bioanalyzer 2100 system to evaluate the quality of the constructed library;

h. using TruSeq PE Cluster Kit v3 cBot HS (Illumina) to generate clusters on cBot, and then the Illumina-Hiseq platform was applied for sequencing.
