# Supplementary material for: The genomic and transcriptome characteristics of lung adenocarcinoma patients with previous breast cancer
Source: BMC Cancer. 2022 Jun 6;22:618. doi: 10.1186/s12885-022-09727-6 (PMC9171992; doi:10.1186/s12885-022-09727-6)
Supplement: Supplementary file 7 — Additional file 7. [file 12885_2022_9727_MOESM7_ESM.docx]

Table S1. Sequencing data quality for the whole exome sequencing.

| Name | ID | Clean reads | Clean data (Gb) | Mapped ratio (%) | Duplicated ratio (%) | On-target ratio (%) | Mean coverage | Q20 (%) | Q30 (%) |
| --- | --- | --- | --- | --- | --- | --- | --- | --- | --- |
| QY | 2000808-T | 156872275 | 23.53 | 99.98 | 21.98 | 63.86 | 144.39 | 97.36 | 92.74 |
| QY | 2000808-N | 85873687 | 12.88 | 99.99 | 20.81 | 71.12 | 77.76 | 97.91 | 94.27 |
| HYH | 2001331-N | 119365623 | 17.9 | 99.97 | 20.79 | 63.33 | 111.76 | 97.11 | 92.27 |
| HYH | 2001331-T | 105367407 | 15.81 | 99.98 | 20.22 | 64.27 | 100.71 | 97.27 | 92.59 |
| LHY | 2001347-N | 104440849 | 15.67 | 99.98 | 20.94 | 63.33 | 98.76 | 97.04 | 92.09 |
| LHY | 2001347-T | 86423318 | 12.96 | 99.98 | 21.14 | 70.47 | 79.19 | 97.79 | 93.90 |
| HP | 2001364-N | 90126278 | 13.52 | 99.98 | 20.29 | 69.99 | 83.68 | 97.80 | 94.00 |
| HP | 2001364-T | 109887513 | 16.48 | 99.98 | 21.01 | 69.83 | 103.02 | 97.73 | 93.73 |
| ZXH | 2001589-N | 110552319 | 16.58 | 99.98 | 20.64 | 62.85 | 105.03 | 96.72 | 91.27 |
| ZXH | 2001589-T | 85991275 | 12.9 | 99.99 | 20.62 | 70.46 | 80.89 | 97.87 | 94.10 |
| HSQ | 2001704-T | 96716217 | 14.51 | 99.99 | 20.95 | 78.85 | 95.96 | 98.16 | 94.79 |
| HSQ | 2001704-N | 74807036 | 11.22 | 99.99 | 19.22 | 79.11 | 76.83 | 98.08 | 94.55 |
| CZQ | 2001705-T | 81132704 | 12.17 | 99.98 | 19.77 | 80.19 | 83.81 | 98.13 | 94.74 |
| CZQ | 2001705-N | 104020432 | 15.6 | 99.98 | 22.08 | 81.02 | 104.33 | 98.11 | 94.70 |
| XBQ | 2002120-T | 81240872 | 12.19 | 99.98 | 19.96 | 79.87 | 81.7 | 98.11 | 94.57 |
| XBQ | 2002120-N | 100083520 | 15.01 | 99.99 | 21.05 | 72.16 | 97.5 | 97.81 | 93.93 |
| ZXL | 2002259-T | 86495596 | 12.97 | 99.99 | 20.09 | 80.67 | 88.62 | 98.25 | 95.00 |
| ZXL | 2002259N | 105927496 | 15.89 | 99.98 | 21.11 | 69.05 | 103.64 | 97.43 | 92.99 |
| HXC | 2002297-T | 109536444 | 16.43 | 99.98 | 22.20 | 71.71 | 106.84 | 97.67 | 93.58 |
| HXC | 2002297-N | 87452540 | 13.12 | 99.98 | 21.36 | 70.54 | 84.57 | 97.65 | 93.58 |
| HZL | 2001631-T | 90719928 | 13.61 | 99.98 | 20.44 | 70.55 | 85.3 | 97.72 | 93.78 |
| HZL | 2001631-N | 133385114 | 20.01 | 99.97 | 21.86 | 63.75 | 125.44 | 97.04 | 92.14 |
| HSZ | 2001701-T | 94115756 | 14.12 | 99.99 | 20.34 | 70.60 | 88.46 | 97.86 | 94.09 |
| HSZ | 2001701-N | 125765421 | 18.86 | 99.98 | 19.71 | 66.26 | 122.65 | 97.42 | 92.97 |
| SXH | 2001703-N | 73823345 | 11.07 | 99.99 | 18.03 | 78.30 | 76.33 | 98.20 | 94.82 |
| SXH | 2001703-T | 75317809 | 11.3 | 99.98 | 20.69 | 80.02 | 76.34 | 98.16 | 94.77 |
| YY | 2002031-N | 127391647 | 19.11 | 99.98 | 22.21 | 67.99 | 123.67 | 97.37 | 92.90 |
| YY | 2002031-T | 100736809 | 15.11 | 99.98 | 21.09 | 79.96 | 101.52 | 98.13 | 94.76 |
| YJH | 2002191-N | 98488231 | 14.77 | 99.98 | 20.51 | 70.17 | 95.93 | 97.58 | 93.30 |
| YJH | 2002191-T | 88916110 | 13.34 | 99.99 | 20.34 | 80.23 | 90.61 | 98.22 | 94.86 |
| XJR | 2002298-T | 94654724 | 14.2 | 99.98 | 21.89 | 68.96 | 95.07 | 97.32 | 92.77 |
| XJR | 2002298-N | 83417988 | 12.51 | 99.98 | 21.32 | 72.20 | 80.16 | 97.82 | 93.91 |
| WSL | 2002388-T | 115581176 | 17.34 | 99.98 | 22.78 | 69.57 | 110.4 | 97.71 | 93.60 |
| WSL | 2002388-N | 75121483 | 11.27 | 99.99 | 20.96 | 71.29 | 72.86 | 97.80 | 93.79 |
| WGY | 2002414-T | 72666253 | 10.9 | 99.99 | 19.11 | 72.22 | 72.78 | 97.80 | 93.86 |
| WGY | 2002414-N | 88633473 | 13.3 | 99.99 | 20.86 | 72.56 | 88.96 | 97.76 | 93.73 |
| YXH | 2002560-T | 79984280 | 12 | 99.99 | 19.83 | 72.66 | 78.12 | 97.96 | 94.26 |
| YXH | 2002560-N | 121648466 | 18.25 | 99.98 | 21.39 | 67.94 | 118.82 | 97.31 | 92.69 |
| CLJ | 2002561-T | 131410205 | 19.71 | 99.98 | 22.64 | 67.21 | 127.17 | 97.37 | 92.88 |
| CLJ | 2002561-N | 104315726 | 15.65 | 99.98 | 21.99 | 68.39 | 102.73 | 97.36 | 92.86 |

T: tumor; N: normal pulmonary tissue.

Ttable S2. Sequencing data quality for the RNA-seq.

| Name | ID | Clean reads | Total mapped（%） | Uniquely mapped（%） | Duplicated ratio（%） | Q20% | Q30% | OD 260/280 |
| --- | --- | --- | --- | --- | --- | --- | --- | --- |
| QY | 2000808 | 16635800 | 97.24 | 88.63 | 11.37 | 97.88 | 93.99 | 1.90 |
| HYH | 2001331 | 51802165 | 98.34 | 88.12 | 11.88 | 98.44 | 95.25 | 1.92 |
| LHY | 2001347 | 11834911 | 94.54 | 81.39 | 18.61 | 98.24 | 95.32 | 1.92 |
| HP | 2001364 | 73612182 | 98.26 | 87.96 | 12.04 | 98.29 | 94.87 | 1.75 |
| ZXH | 2001589 | 60714438 | 97.88 | 86.52 | 13.48 | 98.40 | 95.14 | 1.97 |
| HSQ | 2001704 | 44091412 | 98.30 | 89.00 | 11.00 | 98.24 | 94.74 | 1.95 |
| CZQ | 2001705 | 54826726 | 98.36 | 89.20 | 10.80 | 98.34 | 94.98 | 1.75 |
| XBQ | 2002120 | 58801576 | 98.49 | 89.10 | 10.90 | 98.39 | 95.08 | 1.98 |
| ZXL | 2002259 | 56483143 | 98.03 | 88.17 | 11.83 | 98.26 | 94.75 | 1.87 |
| HXC | 2002297 | 43987880 | 98.18 | 88.17 | 11.83 | 98.47 | 95.37 | 1.95 |
| HZL | 2001631 | 71991576 | 98.42 | 88.83 | 11.17 | 98.37 | 95.06 | 1.92 |
| HSZ | 2001701 | 51690855 | 98.37 | 88.20 | 11.80 | 98.43 | 95.19 | 1.93 |
| SXH | 2001703 | 42097270 | 97.92 | 88.28 | 11.72 | 98.27 | 94.90 | 1.94 |
| XJR | 2002298 | 63398266 | 98.35 | 88.48 | 11.52 | 98.24 | 94.69 | 1.93 |
| WSL | 2002388 | 35607632 | 98.27 | 88.03 | 11.97 | 98.17 | 94.58 | 1.92 |
| WGY | 2002414 | 42235520 | 98.14 | 88.88 | 11.12 | 98.25 | 94.75 | 1.97 |
| YXH | 2002560 | 49027618 | 98.10 | 87.57 | 12.43 | 98.32 | 94.93 | 1.95 |
| CLJ | 2002561 | 70140194 | 98.34 | 88.40 | 11.60 | 98.29 | 94.79 | 1.95 |

Table S3. Results of somatic copy number variation analysis.

| Name | ID | Amplified genes | Deleted genes |
| --- | --- | --- | --- |
| QY | 2000808 | CDK6、EGFR、FGFR3、HGF、MET、MYC、RICTOR、ROS1 | FLCN、NF2、SMARCA4、STK11、TP53、TSC1 |
| HYH | 2001331 | MCL1、MDM4、NTRK1 | - |
| LHY | 2001347 | - | - |
| HP | 2001364 | - | - |
| ZXH | 2001589 | BRAF、CCND1、CCND3、FGF19、FGF3、FGF4、FGFR3、FOXA1、MCL1、MDM4、MYC、NTRK1、VEGFA | APC、CDKN2A、CDKN2B、PTEN |
| HSQ | 2001704 | - | - |
| CZQ | 2001705 | - | - |
| XBQ | 2002120 | - | - |
| ZXL | 2002259 | - | - |
| HXC | 2002297 | - | - |
| HZL | 2001631 | BRAF | CDKN2A、CDKN2B、INPP4B、PTEN |
| HSZ | 2001701 | MDM4 | - |
| SXH | 2001703 | MCL1、MDM4、NTRK1 | - |
| YY | 2002031 | - | - |
| YJH | 2002191 | - | - |
| XJR | 2002298 | LRP1B、RICTOR | - |
| WSL | 2002388 | EGFR、RICTOR | - |
| WGY | 2002414 | FOXA1、NRAS、RICTOR | - |
| YXH | 2002560 | - | PTEN |
| CLJ | 2002561 | CCNE1、MCL1、MDM4 | TP53 |

Table S4. The enrich and down-regulated hallmarks gene set.

| Name | Size | ES | NES | *P*-value | *P*.adjust value | FDR *q*-value |
| --- | --- | --- | --- | --- | --- | --- |
| HALLMARK_TNFA_SIGNALING_VIA_NFKB | 10 | -0.527 | -1.706 | 0.026 | 0.306 | 0.295 |

| Name | Size | ES | NES | *P* value | *P*.adjust value | FDR *q*-value |
| --- | --- | --- | --- | --- | --- | --- |
| Vascular process in circulatory system | 113 | 0.541 | 2.069 | 0.002 | 0.013 | 0.008 |
| Regulation of endothelial cell migration | 122 | 0.520 | 2.018 | 0.002 | 0.013 | 0.008 |
| Regulation of wound healing | 104 | 0.522 | 1.963 | 0.002 | 0.013 | 0.008 |
| Regulation of system process | 401 | 0.443 | 1.949 | 0.001 | 0.013 | 0.008 |
| G protein-coupled receptor signaling pathway, coupled to cyclic nucleotide second messenger | 101 | 0.517 | 1.942 | 0.002 | 0.013 | 0.008 |
| Heart morphogenesis | 191 | 0.468 | 1.912 | 0.002 | 0.013 | 0.008 |
| Organ growth | 129 | 0.484 | 1.884 | 0.002 | 0.013 | 0.008 |
| Blood circulation | 374 | 0.423 | 1.856 | 0.001 | 0.013 | 0.008 |
| Circulatory system process | 381 | 0.422 | 1.852 | 0.001 | 0.013 | 0.008 |
| Regulation of blood pressure | 118 | 0.480 | 1.842 | 0.002 | 0.013 | 0.008 |
| Negative regulation of growth | 165 | 0.457 | 1.839 | 0.002 | 0.013 | 0.008 |
| Angiogenesis | 349 | 0.421 | 1.831 | 0.001 | 0.013 | 0.008 |
| Positive regulation of epithelial cell proliferation | 132 | 0.464 | 1.822 | 0.002 | 0.013 | 0.008 |
| Regulation of epithelial cell migration | 163 | 0.448 | 1.802 | 0.002 | 0.013 | 0.008 |
| Endothelial cell migration | 156 | 0.451 | 1.801 | 0.002 | 0.013 | 0.008 |
| Blood vessel morphogenesis | 423 | 0.407 | 1.799 | 0.001 | 0.013 | 0.008 |
| Regulation of response to wounding | 125 | 0.460 | 1.796 | 0.002 | 0.013 | 0.008 |
| Endothelial cell proliferation | 107 | 0.475 | 1.793 | 0.002 | 0.013 | 0.008 |
| G protein-coupled receptor signaling pathway | 486 | 0.400 | 1.789 | 0.001 | 0.013 | 0.008 |
| Organic hydroxy compound transport | 139 | 0.443 | 1.744 | 0.002 | 0.013 | 0.008 |
| Heart contraction | 197 | 0.422 | 1.740 | 0.001 | 0.013 | 0.008 |
| Negative regulation of cell growth | 108 | 0.457 | 1.734 | 0.002 | 0.013 | 0.008 |
| Positive regulation of synaptic transmission | 110 | 0.455 | 1.732 | 0.002 | 0.013 | 0.008 |
| Circulatory system development | 761 | 0.373 | 1.722 | 0.001 | 0.013 | 0.008 |
| Blood vessel development | 484 | 0.385 | 1.721 | 0.001 | 0.013 | 0.008 |
| Second-messenger-mediated signaling | 232 | 0.412 | 1.719 | 0.001 | 0.013 | 0.008 |
| Fatty acid derivative metabolic process | 101 | 0.458 | 1.718 | 0.002 | 0.013 | 0.008 |
| Fat cell differentiation | 135 | 0.438 | 1.716 | 0.002 | 0.013 | 0.008 |
| Regulation of body fluid levels | 344 | 0.395 | 1.714 | 0.001 | 0.013 | 0.008 |
| Vasculature development | 507 | 0.382 | 1.713 | 0.001 | 0.013 | 0.008 |

Table S5. TOP30 significantly up-regulated gene set in all GO analysis.

Table S6. TOP30 significantly down-regulated gene set in all GO analysis.

| Name | Size | ES | NES | *P* value | *P*.adjust value | FDR *q*-value |
| --- | --- | --- | --- | --- | --- | --- |
| Spindle | 199 | -0.318 | -1.409 | 0.003 | 0.014 | 0.008 |
| Chromosomal region | 210 | -0.391 | -1.740 | 0.003 | 0.014 | 0.008 |
| Condensed chromosome | 117 | -0.470 | -1.923 | 0.003 | 0.014 | 0.008 |
| Chromosome, centromeric region | 113 | -0.487 | -1.975 | 0.003 | 0.014 | 0.008 |
| Chromosomal part | 505 | -0.317 | -1.528 | 0.004 | 0.017 | 0.010 |
| Chromosome | 577 | -0.310 | -1.504 | 0.004 | 0.018 | 0.010 |
| Chromosome segregation | 157 | -0.443 | -1.871 | 0.003 | 0.019 | 0.012 |
| DNA conformation change | 163 | -0.432 | -1.836 | 0.003 | 0.019 | 0.012 |
| Nuclear chromosome segregation | 125 | -0.431 | -1.757 | 0.003 | 0.019 | 0.012 |
| DNA replication | 169 | -0.383 | -1.636 | 0.003 | 0.019 | 0.012 |
| DNA recombination | 158 | -0.381 | -1.608 | 0.003 | 0.019 | 0.012 |
| DNA metabolic process | 595 | -0.286 | -1.406 | 0.005 | 0.025 | 0.016 |
| Chromosome organization | 655 | -0.296 | -1.461 | 0.005 | 0.025 | 0.016 |
| Nuclear division | 200 | -0.337 | -1.468 | 0.006 | 0.030 | 0.019 |
| Ribonucleoprotein complex | 355 | -0.284 | -1.323 | 0.010 | 0.032 | 0.019 |
| Nuclear chromosome | 313 | -0.282 | -1.297 | 0.013 | 0.038 | 0.023 |
| DNA-binding transcription factor activity | 901 | -0.283 | -1.419 | 0.005 | 0.039 | 0.030 |
| DNA-binding transcription factor activity, RNA polymerase II-specific | 863 | -0.289 | -1.451 | 0.005 | 0.039 | 0.030 |
| Negative regulation of cell cycle process | 188 | -0.331 | -1.434 | 0.009 | 0.040 | 0.026 |
| Translation | 288 | -0.299 | -1.372 | 0.010 | 0.042 | 0.027 |
| Chromosome, telomeric region | 100 | -0.365 | -1.447 | 0.015 | 0.044 | 0.026 |
| Telomere organization | 110 | -0.390 | -1.579 | 0.010 | 0.044 | 0.028 |
| DNA repair | 324 | -0.294 | -1.352 | 0.011 | 0.044 | 0.028 |
| Protein-DNA complex assembly | 119 | -0.374 | -1.518 | 0.011 | 0.045 | 0.029 |
| Regulation of cell cycle G2/M phase transition | 128 | -0.355 | -1.457 | 0.011 | 0.045 | 0.029 |
| Organelle fission | 222 | -0.306 | -1.368 | 0.012 | 0.048 | 0.031 |
| Adaptive immune response | 284 | -0.292 | -1.334 | 0.013 | 0.049 | 0.032 |
| Tumor necrosis factor-mediated signaling pathway | 106 | -0.382 | -1.535 | 0.013 | 0.050 | 0.032 |

Table S7. Specific information about the 13 significantly up-expressed genes.

| Name | Description | Log_2_Fold change | *P*.adjust |
| --- | --- | --- | --- |
| EDN1 | Endothelin 1 | 1.603 | 0.041 |
| CAV1 | Caveolin 1 | 2.190 | <0.001 |
| VEGFA | Vascular endothelial growth factor A | 1.236 | 0.008 |
| AGTR2 | Angiotensin II receptor type 2 | 4.125 | 0.002 |
| FGF10 | Fibroblast growth factor 10 | 1.538 | 0.037 |
| WNT3A | Wnt family member 3A | 2.843 | <0.001 |
| DAB2IP | DAB2 interacting protein | 1.380 | <0.001 |
| TEK | TEK receptor tyrosine kinase | 2.354 | <0.001 |
| WNT7A | Wnt family member 7A | 2.737 | <0.001 |
| AGER | Advanced glycosylation end-product specific receptor | 3.693 | <0.001 |
| PPARG | Peroxisome proliferator activated receptor gamma | 1.598 | 0.006 |
| CD36 | CD36 molecule | 1.531 | 0.019 |
| ADCY8 | Adenylate cyclase 8 | 4.206 | <0.001 |
